# Safety and pharmacodynamics of dalazatide, a Kv1.3 channel inhibitor, in the treatment of plaque psoriasis: A randomized phase 1b trial

**DOI:** 10.1371/journal.pone.0180762

**Published:** 2017-07-19

**Authors:** Eric J. Tarcha, Chelsea M. Olsen, Peter Probst, David Peckham, Ernesto J. Muñoz-Elías, James G. Kruger, Shawn P. Iadonato

**Affiliations:** 1 Kineta Inc., Seattle, WA, United States of America; 2 Laboratory for Investigative Dermatology, The Rockefeller University, New York, NY, United States of America; University of Münster, GERMANY

## Abstract

**Background:**

Dalazatide is a specific inhibitor of the Kv1.3 potassium channel. The expression and function of Kv1.3 channels are required for the function of chronically activated memory T cells, which have been shown to be key mediators of autoimmune diseases, including psoriasis.

**Objective:**

The primary objective was to evaluate the safety of repeat doses of dalazatide in adult patients with mild-to-moderate plaque psoriasis. Secondary objectives were to evaluate clinical proof of concept and the effects of dalazatide on mediators of inflammation in the blood and on chronically activated memory T cell populations.

**Methods:**

Patients (*n* = 24) were randomized 5:5:2 to receive dalazatide at 30 mcg/dose, 60 mcg/dose, or placebo twice weekly by subcutaneous injection (9 doses total). Safety was assessed on the basis of physical and neurological examination and laboratory testing. Clinical assessments included body-surface area affected, Psoriasis Area and Severity Index (PASI), and investigator and patient questionnaires.

**Results:**

The most common adverse events were temporary mild (Grade 1) hypoesthesia (*n* = 20; 75% placebo, 85% dalazatide) and paresthesia (*n* = 15; 25% placebo, 70% dalazatide) involving the hands, feet, or perioral area. Nine of 10 patients in the 60 mcg/dose group had a reduction in their PASI score between baseline and Day 32, and the mean reduction in PASI score was significant in this group (*P* < 0.01). Dalazatide treatment reduced the plasma levels of multiple inflammation markers and reduced the expression of T cell activation markers on peripheral blood memory T cells.

**Limitations:**

The study was small and drug treatment was for a short duration (4 weeks).

**Conclusion:**

This study indicates that dalazatide is generally well tolerated and can improve psoriatic skin lesions by modulating T cell surface and activation marker expression and inhibiting mediators of inflammation in the blood. Larger studies of longer duration are warranted.

## Introduction

Dalazatide (previously referred to as ShK-186 and SL5) is a 37-amino acid synthetic peptide that is a specific inhibitor of the voltage-gated Kv1.3 potassium channel [1]. It is a derivative of ShK, which was originally isolated from the venom of the sea anemone *Stichodactyla helianthus* [2]. Human T cells rely on two potassium channels, Kv1.3 and KCa3.1 to maintain the calcium signaling required for their activation and differentiation, by maintaining a balance between calcium influx and potassium efflux. Chronically activated memory T cells that rely on upregulation of Kv1.3 expression for their effector function are sensitive to blockade by Kv1.3 inhibitors. These chronically activated memory T cells are key mediators of numerous autoimmune diseases, including psoriasis [3,4]. Other T cell subsets that upregulate the calcium-activated KCa3.1 channel upon activation are insensitive to blockade by Kv1.3 inhibitors [5]. It has been demonstrated that in the effector memory T (T_EM_) cell population, the degree of Kv1.3 expression is a measure of cell activation, with greater Kv1.3 numbers/cell following repeated stimulation and that the channel is required for the maintenance of the T_EM_ cell phenotype [6]. These properties make Kv1.3 channels on T lymphocytes an attractive therapeutic target for autoimmune diseases (reviewed recently in [7]). Recent data suggest that the degree of Kv1.3 dependence is mediated by antigen exposure history, that chronically activated autoreactive T cells are primarily dependent on Kv1.3, and that conversion to Kv1.3 dependency is stable [8]. Other lymphocytes, including class-switched memory B cells also depend upon Kv1.3 for activation, however their sensitivity to blockade by Kv1.3 inhibitors is still being explored [9]. While various other cells of the immune system also express Kv1.3 channels, those cells express numerous K^+^ channels and are not sensitive to blockade by Kv1.3 inhibitors due to channel redundancy [10]. In vivo studies with dalazatide in a delayed-type hypersensitivity model have shown that drug treatment inhibited the DTH response by suppressing T_EM_ cells, but had no effect on naïve or central memory T cells [11]. In addition, animals chronically treated with dalazatide are able to clear viral and bacterial infections similar to vehicle treated animals, while those treated with dexamethasone, which broadly suppresses the immune response have a significantly delayed clearance rate, further supporting the specific and immune sparing role of dalazatide [11].

Psoriasis is a chronic autoimmune disease that causes raised, scaly patches on the skin. It is one of the most frequent human skin diseases, affecting about 3% of the United States population [12]. Plaque psoriasis is the most common variant of the disease, affecting about 90% of patients. Treatment of mild-to-moderate disease, which affects about 80% of patients, consists of topical medications typically containing corticosteroids or vitamin D analogs [13]. Phototherapy, involving exposure to UVA or UVB light alone or in combination with a photosensitizer, is recommended for moderate-to-severe psoriasis [14]. Systemic medications, including methotrexate, cyclosporine, acitretin, azathioprine, and sulfasalazine, are used to treat psoriasis that is too extensive for, or refractory to, topical or UV therapy [15, 16]. However, these medications have significant dose-limiting toxicities. Several biologic drugs, particularly TNF, IL-17 and IL-23 inhibitors, have been approved or are in development for the treatment of moderate-to-severe psoriasis [17, 18, 19]. Despite the variety of treatments available, in a study of over 5,000 psoriasis patients, over 50% were dissatisfied with their treatment [20].

Dendritic cells and T_EM_ cells are central players in the development of psoriatic skin lesions. These cells produce cytokines that increase the migration of inflammatory cells into the skin and that stimulate keratinocytes to proliferate, resulting in epidermal hyperplasia [21]. Psoriatic plaques are enriched with T_EM_ cells that express the Kv1.3 channel [22] as well as cutaneous lymphocyte-associated antigen (CLA), which guides T cells to the skin [23]. Inhibition of the Kv1.3 channel suppresses the activation and proliferation of T_EM_ cells, and there is considerable interest in evaluating Kv1.3 channel blockers, such as dalazatide, for the treatment of psoriasis and other autoimmune diseases [24, 25].

This Phase 1B study evaluated two dose levels of dalazatide given subcutaneously to patients with mild-to-moderate plaque psoriasis. The primary objectives were to evaluate the safety, tolerability, and immunogenicity of repeat doses of the drug. Secondary objectives were to evaluate clinical proof of concept and pharmacokinetic and pharmacodynamic parameters, including the effects of dalazatide treatment on plasma protein analyte levels and T cell populations. It is reported here that dalazatide treatment was well tolerated, without serious adverse events, and reduced mediators of inflammation in the blood and decreased the expression of T cell activation markers. This is the first Kv1.3 inhibitor to be tested in clinical trials for autoimmune diseases.

## Methods

### Trial registration

ClinicalTrials.gov NCT02435342

### Patients

Eligible patients were adult men and women (age 18 to 65 years; weight 50 to 100 kg) with active plaque psoriasis (≥ 3% body-surface area affected). Inclusion criteria included psoriatic plaques of at least 2 × 2 cm with Target Lesion Investigator Global Assessment scores ≥ 3 that were not located on the face, scalp, groin, genitals, folds, palms, or soles. Exclusion criteria included current drug-induced or aggravated psoriasis, the use of systemic or topical medications or UVA or UVB therapy within 4 weeks of baseline, the presence or history of paresthesia or neuropathy, a history of cancer requiring systemic chemotherapy or radiation, or the presence of an acute infection within 7 days of baseline. Women of child-bearing potential were excluded unless they agreed to use contraception. An Institutional Review Board approved the protocol (S1 Protocol) and all patients provided written informed consent.

### Study design

**Fig 1:**
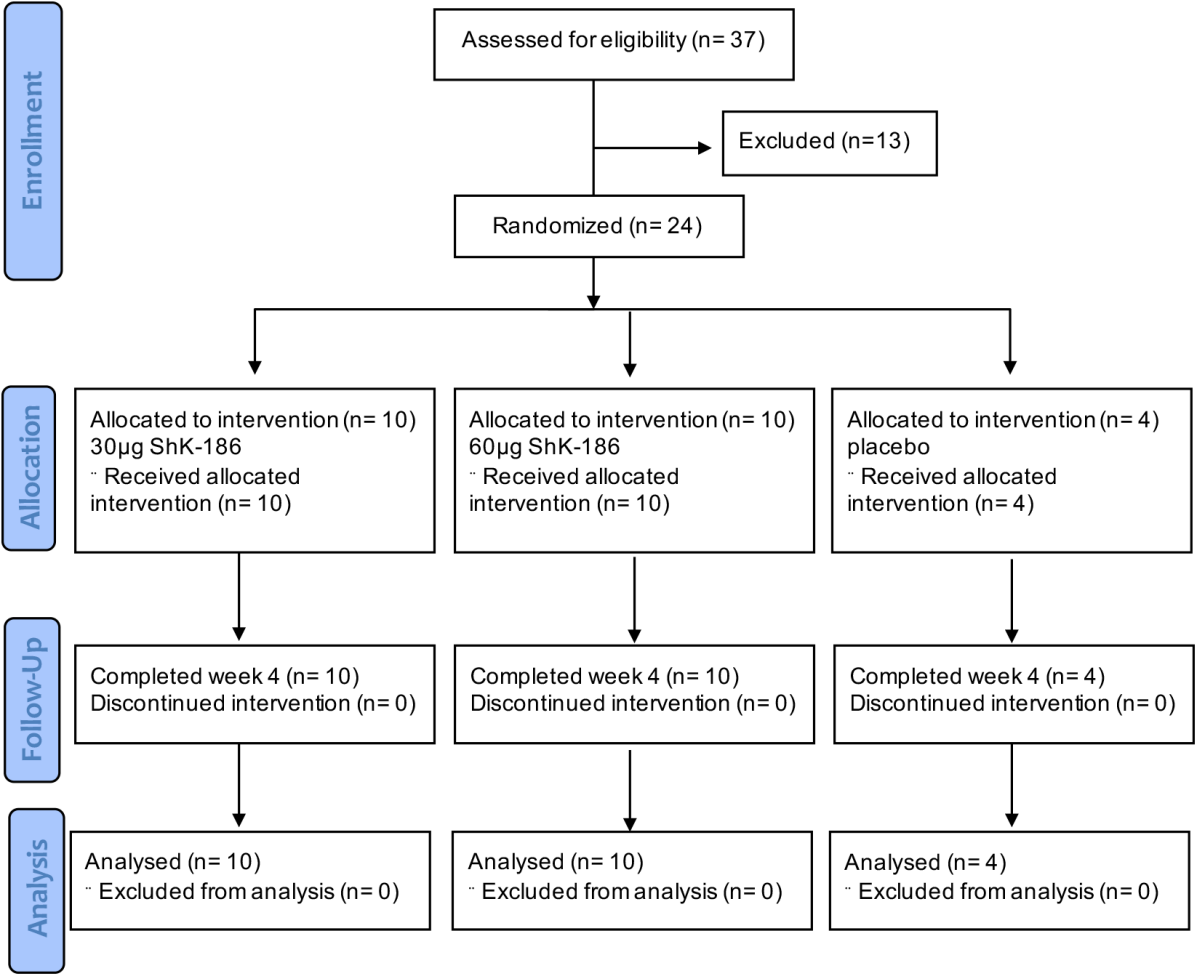
Consort flow diagram. Graphical outline of the design and conduct of the study.

The trial was a double-blind (subject and investigator), placebo-controlled study conducted at two Canadian centers. The trial was conducted under an investigational new drug application approved by the US Food and Drug Administration and the Institutional Review Board, and was performed in accordance with the Declaration of Helsinki and with the applicable International Conference on Harmonization Guidelines. A total of 24 patients with active plaque psoriasis were randomized 5:5:2 to receive dalazatide at 30 mcg/dose, 60 mcg/dose, or placebo twice weekly by subcutaneous injection into the abdominal fat pad (9 doses total) (Fig 1; S1 CONSORT Checklist and S1 Protocol). The study randomization was prepared by Innovaderm using Medrio’s Electronic Data Capture system (San Francisco, CA). The sample size chosen for this study was based upon precedent set by other studies of a similar nature and was not based on power calculations. Patients were screened to assess their eligibility to enter the study within 30 days of the Day 1 (first-dose) visit. Dose administration and evaluation were on Days 1, 4, 8, 11, 15, 18, 22, 25, and 29. Additional follow-up evaluations were on Days 32, 43, and 57. This was a double-blind, placebo-controlled study. As such, except for the specifically designated unblinded study site clinical and ancillary staff, the Investigators and remaining study site clinical staff were blinded as to treatment. The unblended staff members were not involved in any on-study data collection. Patients were enrolled in the study beginning October 2014 and the last patient last visit was in March 2015.

### Safety assessments

Safety was assessed on the basis of vital signs (weight, blood pressure, pulse, oral temperature, and respiratory rate) and physical examination on all dose administration and evaluation days. A neurological exam was performed on Days 1 and 57. Laboratory testing (urinalysis, serum chemistry, and hematology) was performed on Days −1, 8, 15, 22, 25, and 57.

### Immunogenicity assessment

Serum samples were collected from all patients on Days −1, 15, 29, and 57 and analyzed for dalazatide anti-drug antibodies (ADA) by a non-GLP ADA ELISA, utilizing derail dilutions of anti-dalazatide monoclonal antibody P3B3 in pooled human serum as the primary antibody and a horseradish peroxidase conjugated recombinant protein A/G secondary antibody. Optical densities for each well were obtained using a spectrophotometer set at 450 nm wavelength. Samples with readings above the ELISA cutoff point were analyzed by using an immune-depletion assay to assess antibody specificity. Samples with a ≥50% reduction of signal after incubation with dalazatide were considered to be positive for specific anti-dalazatide antibodies.

### Pharmacokinetic assessments

Pharmacokinetic (PK) analysis, including serum concentration, maximum concentration (C_max_), time to reach C_max_ (t_max_), and area under the curve (AUC) from time 0 to the last measurable time point calculated using the linear trapezoidal rule, were assessed as secondary endpoints for all patients. Plasma samples were obtained on Day 1 (predose, 0.083, 0.25, 0.5, 1, 2, and 4 hour), Day 15 (predose), and Day 29 (predose, 0.083, 0.25, 0.5, 1, 2, 4, hour). The PK samples were analyzed for dalazatide concentration by using a qualified high-sensitivity, two-dimensional liquid chromatography tandem mass spectrometry (2D-LC-MS/MS)-based assay. The lower limit of quantification (LLOQ) for the qualified method was 75 pg/ml. The lower limit of detection (LLOD) of the assay based on the standard curve was set as 10 pg/ml, and reported values for samples between the LLOQ and LLOD were extrapolated from the assay standard curve. Any samples below the LLOD were reported as below the limit of quantitation.

### Efficacy assessments

Clinical response was assessed on Days 1, 15, 32, and 57 by body-surface area (BSA) affected (0 to 100% as evaluated by a dermatologist), Dermatology Life Quality Index (DLQI; 10-question patient questionnaire), Psoriasis Area and Severity Index (PASI; a measure that combines scores for erythema, induration, desquamation, and BSA affected; ranging from 0 for no disease to 72 for the most severe disease), Psoriasis Disability Index (PDI; 15-question patient questionnaire), Investigator’s Global Assessment (0, clear; 1, almost clear; 2, mild; 3, moderate; or 4, severe), Patient Global Assessment (ranging from 0, clear to 10, severe), and Target Lesion Investigator Global Assessment (TLIGA; target lesion erythema, induration, and scaling on a scale of 0, clear; 1, almost clear; 2, mild; 3, moderate; or 4, severe).

### Pharmacodynamic assessments

Blood samples were collected on Days −7, −1, 1, 15, 29, 32, 43, and 57. Blood was collected into two 8-mL CPT Vacutainer tubes (BD Biosciences) and centrifuged at 1500–1800 x g for 20 minutes at room temperature within 2 hours of blood collection. Plasma (5–6 mL) was harvested to 6 aliquots, frozen on dry ice, and stored at -80 deg C. A Luminex multiplex bead-array immunoassay was performed on blood samples from the placebo and 60 mcg dose groups from Days 1 and 32 to measure the level of 32 plasma protein analytes. A single-molecule-counting Erenna Immunoassay System (Singulex) was used to measure IL-17A. *Ex vivo* flow cytometry analysis was performed to characterize expression of activation markers HLA-DR, KI67 following culture in media alone or CD40L following stimulation with 0.2 nM phorbol myristate acetate (PMA) and ionomycin (250 ng/ml) for 6 hours. Activation marker expression was evaluated on subpopulations of T cell populations. T cells were identified by gating on CD2^+^ cells. Skin-homing T cells were identified by gating on CD2^+^ CLA^+^ cells. CD4^+^ and CD8^+^ subsets were further divided into effector memory (T_EM_; CD45RO^+^ CCR7−), central memory (T_CM_; CD45RO^+^ CCR7^+^), and regulatory T cells (CD4^+^ CD25^+^ FOXP3^+^) and then analyzed for cell surface and activation markers.

### Statistical analysis

To compare plasma markers of inflammation in the 60 mcg/dose dalazatide and placebo groups, a two-tailed, unpaired *t*-test was performed on the percent change of inflammatory markers levels in the plasma from baseline (Day 1) to Day 32 of the study. The effect of Dalazatide treatment on the expression of T cell activation markers was determine by using a one-tailed, unpaired *t*-test comparing the percent change from baseline to Day 29 of 60 mcg/dose dalazatide and placebo groups. Statistical analyses were performed using GraphPad Prism 5.04 for Windows (GraphPad Software, La Jolla California; www.graphpad.com).

## Results

### Patient characteristics

Of the 37 patients who were screened, 24 with active plaque psoriasis were enrolled and treated with blinded study drug. Four patients were randomized to placebo, 10 to the 30 mcg/dose group, and 10 to the 60 mcg/dose group. All 24 enrolled patients received all 9 planned doses of study drug or placebo and completed the study. Although there was some variability, baseline demographics and clinical disease characteristics were generally similar across treatment groups (Table 1). The age of patients in this study ranged from 30 to 62 years, 46% were female, and 92% were white. The baseline disease activity was typically mild to moderate in all three treatment groups with the groups receiving active treatment trending toward more activity in the PASI, DLQI, and PDI. The mean baseline PASI score was 6.3 (median 6.15) with a mean BSA of 4.25 (median 4.0).

**Table 1:** Patient demographics and baseline disease characteristics.

Characteristic	Placebo (*n* = 4)	Dalazatide 30 mcg/dose (*n* = 10)	Dalazatide 60 mcg/dose (*n* = 10)
Age in years, median (min, max)	58 (41, 61)	51.5 (30, 62)	43.5 (31, 56)
Female, *n* (%)	2 (50)	5 (50)	4 (40)
Race, *n* (%)			
Asian	0	0	1 (10)
Black/African American	0	0	1 (10)
White	4 (100)	10 (100)	8 (80)
Hispanic/Latino ethnicity, *n* (%)	0	0	0
Body weight in kg, median (min, max)	82.2 (64.9, 94.2)	82.1 (67.5, 93.9)	88.8 (49.2^a^, 93.5)
TLIGA^b^ score, median (min, max)	3 (3, 3)	3 (3, 3)	3 (3, 4)
PASI^c^ score, median (min, max)	4.6 (3.8, 5.9)	7.2 (3.4, 11.6)	6.4 (4.6, 9.2)
IGA^d^ score, median (min, max)	3 (3, 3)	3 (3, 3)	3 (3, 4)
%BSA^e^, median (min, max)	3.0 (3.0, 3.5)	4.5 (3.0, 13.0)	4.0 (3.0, 7.0)
DLQI^f^ score, median (min, max)	4.5 (1, 10)	7 (0, 14)	8 (1, 23)
PDI^g^ score, median (min, max)	4 (0, 6)	3.5 (0, 16)	10 (0, 34)

^a^ Subject 03–006 did not meet the minimum weight criterion but was granted a waiver to enroll in the study.

^b^ Target lesion investigator global assessment (TLIGA) scores can range from 0 to 4, with higher scores indicating more severe erythema, induration, and scaling.

^c^ Psoriasis Area and Severity Index (PASI) scores can range from 0 to 72, with higher scores indicating more severe disease.

^d^ Investigator Global Assessment (IGA) scores can range from 0 to 4, with higher score indicating more severe disease.

^e^ Percent Body Surface Area (%BSA) scores can range from 0 to 100, with higher score indicating more affected area.

^f^ Dermatology Life Quality Index (DLQI) scores can range from 0 to 30, with higher score indicating more impaired quality of life during the preceding week.

^g^ Psoriasis Disability Index (PDI) scores can range from 0 to 45, with higher score indicating more extensive disability over the preceding 4 weeks.

### Safety, tolerability, and immunogenicity

Dalazatide was generally well tolerated by all patients and no serious adverse events or discontinuation of treatment occurred (Table 2). The most common adverse events were hypoesthesia (*n* = 20; 75% placebo, 85% dalazatide) and paresthesia (*n* = 15; 25% placebo, 70% dalazatide). Paresthesia most often involved the hands, feet, or perioral area. Hypoesthesia or paresthesia spontaneously resolved after each dose, typically within 12 hours. All events of hypoesthesia and paresthesia were of mild severity (Grade 1), and all were reported as resolved. The proportion of patients with hypoesthesia and paresthesia was not elevated in the 60 mcg/dose group relative to patients who received 30 mcg/dose.

**Table 2:** Summary of treatment-emergent adverse events.

Adverse event (AE)	Placebo (*n* = 4)	Dalazatide 30 mcg/dose (*n* = 10)	Dalazatide 60 mcg/dose (*n* = 10)
Patients with any AE, n (%)	4 (100)	10 (100)	10 (100)
Patients with related AE^a^, *n* (%)	2 (50)	10 (100)	10 (100)
Patients with severe AE, *n* (%)	0	0	0
Discontinuation due to AE, *n* (%)	0	0	0
Related AEs reported in ≥2 patients			
Hypoesthesia	2 (50)	9 (90)	8 (80)
Paresthesia	1 (25)	7 (70)	7 (70)
Muscle spasms	0	4 (40)	3 (30)
Injection site pain	0	1 (10)	3 (30)
Sensitivity of teeth	0	0	4 (40)
Chills	0	1 (10)	2 (20)
Feeling hot	0	0	2 (20)
Muscle contractions (involuntary)	0	0	2 (20)

^a^ AEs with a relationship judged by the Investigator to be 'possible', 'probable', or 'definite' were considered related.

Other adverse events that were reported included nasopharyngitis (*n* = 8; 50% placebo, 30% dalazatide), muscle spasms (*n* = 7; 0% placebo, 35% dalazatide), headache (*n* = 4; 25% placebo, 15% dalazatide), and sensitivity of teeth (*n* = 4; 0% placebo, 20% dalazatide). No significant laboratory abnormalities or changes in vital signs were observed over the course of the study. Overall, the majority of the adverse events were of mild-to-moderate severity. One event was rated as severe for a patient in the placebo group who experienced neutropenia that was considered unrelated to treatment. None of the patients developed antibodies to dalazatide. Two patients had antibody titers above the cutoff point in the initial ELISA, but a subsequent antibody specificity assay demonstrated that the antibodies were not specific to dalazatide.

### Pharmacokinetics

**Fig 2:**
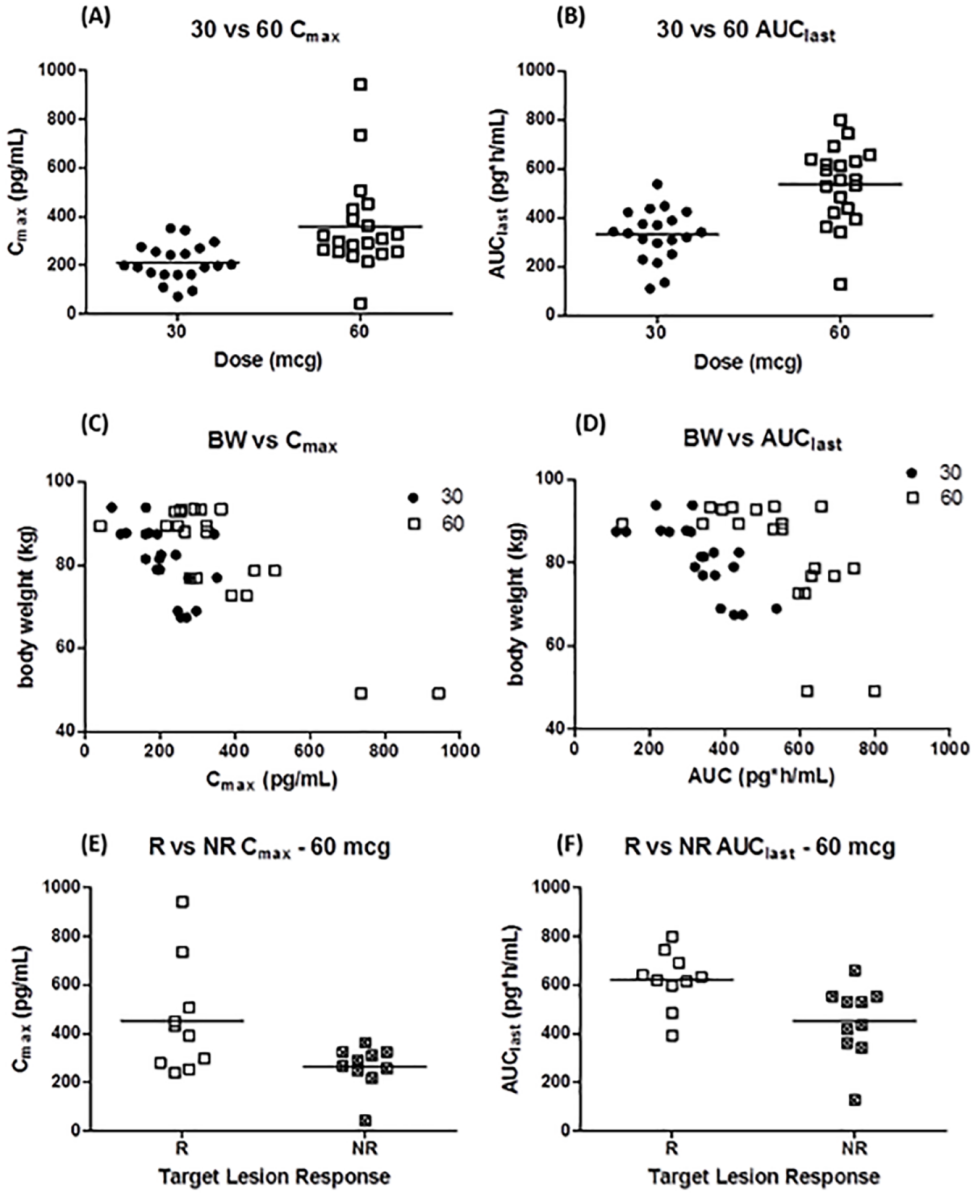
Pharmacokinetic analyses. Individual values for total drug exposure based on mean C_max_ (A) and AUC_last_ (B) for both the 30mcg/dose group (closed circles) and the 60mcg/dose group (open squares). Individual drug exposure levels correlated with body weight for C_max_ (C) and AUC_last_ (D). Individual drug exposure levels from the 60 mcg/dose group separated by those who had an improvement in target lesion response, responders (open square), vs those who did not yet show improvement, nonresponders (hatched square) based on C_max_ (E) and AUC_last_ (F).

This study evaluated the effects of dose level, gender, body weight, and repeated dosing on PK parameters. Additionally, the relationship between exposure and TLIGA response was investigated. PK parameters were evaluated after a single dose (Day 1) and after repeat dosing (Day 29) (Table 3). The dalazatide concentrations from all patients in the placebo group were below the limit of quantitation. The median T_max_ of dalazatide was approximately 0.3 hour post-dose for both dose levels. The T_1/2_ was not computed since data were only collected through the 4-hour time point. Dalazatide exposure based on mean C_max_ and AUC_last_ increased in a less than dose-proportional manner on Day 1 and were approximately dose proportional on Day 29 in the 30 and 60 mcg/dose groups. Individual values for total drug exposure based on mean C_max_ and AUC_last_ are shown in Fig 2A and 2B. Individual exposures for both Day 1 and Day 29 are included, as little or no accumulation was observed. After single (Day 1) or repeat (Day 29) dosing of 30 or 60 mcg, there was no obvious trend in drug exposure based on gender (data not shown). After single or repeat dosing of 30 or 60 mcg, there appeared to be a trend for C_max_ (Fig 2C) and AUC_last_ (Fig 2D) values to decrease with increasing body weight. Evaluation of TLIGA data showed that 5 of the 10 patients in the 60 mcg/dose group had at least a 1 unit improvement in TLIGA at Day 32 (i.e., were responders). Among patients in that dose group, the mean total drug exposure was higher among the TLIGA responders, suggesting a trend toward better response with higher total drug exposure (Fig 2E and 2F).

**Table 3:** Summary of pharmacokinetic parameters at Day 1 and Day 29.

**Day 1**	**Dalazatide** **30 mcg/dose** **(*n* = 10)**	**Dalazatide** **60 mcg/dose** **(*n* = 10)**
T_max_ in hours, median (range)	0.258 (0.250, 0.533)	0.250 (0.0833, 0.517)
C_max_ in pg/ml, mean (standard deviation, SD)	85.3 (39.8)	137 (80.2)
AUC_last_ in h*pg/ml, mean (SD)	55.3 (29.2)	78.4 (39.1)
**Day 29**		
T_max_ in hours, median (range)	0.250 (0.0833, 0.500)	0.250 (0.0833, 0.500)
C_max_ in pg/ml, mean (SD)	76.7 (32.5)	137 (56.0)
AUC_last_ in h*pg/ml, mean (SD)	50.6 (23.3)	88.7 (36.3)

### Clinical proof of concept

**Fig 3:**
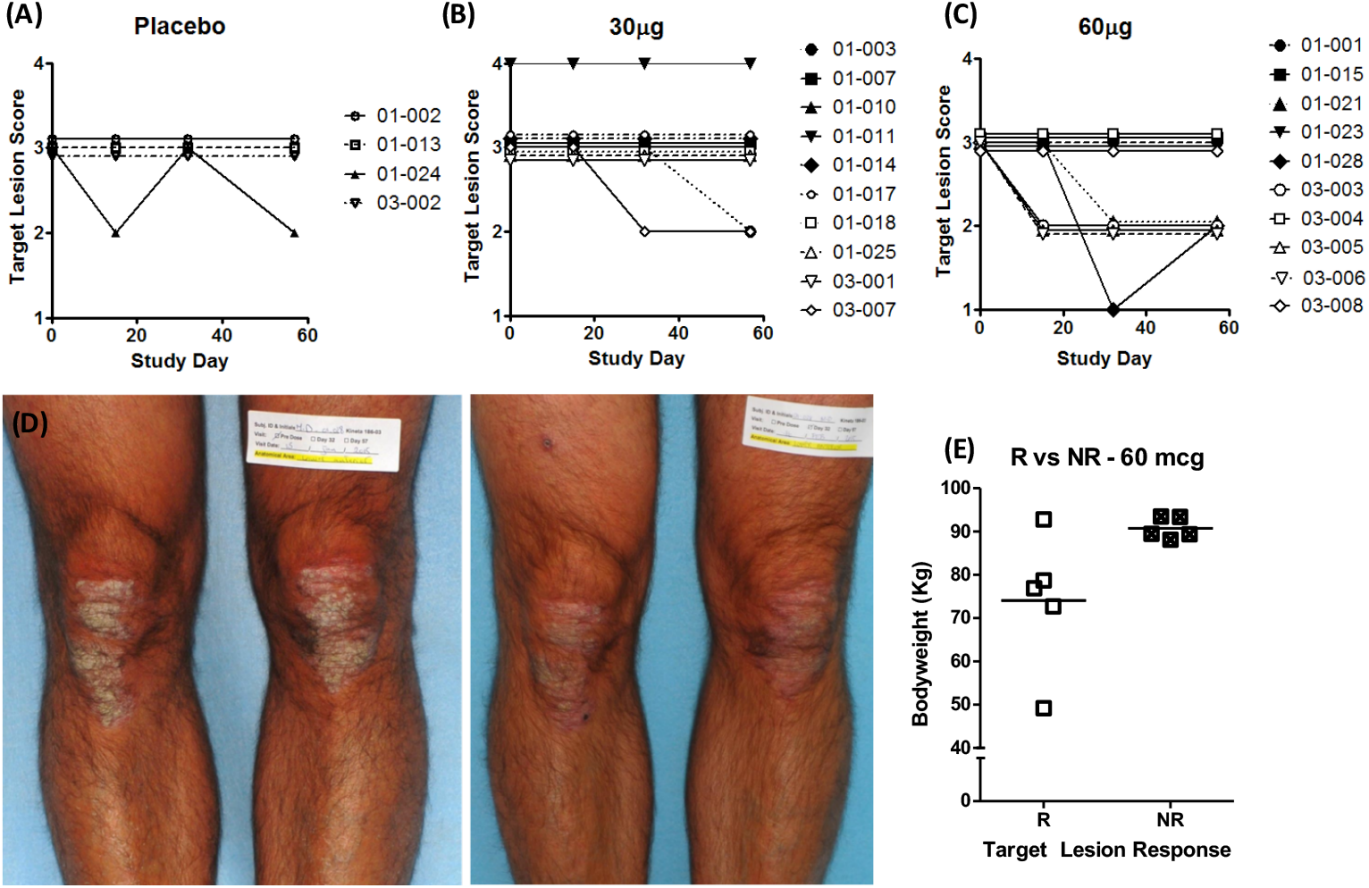
Individual TLIGA scores over time. Placebo group (A), 30 mcg/dose group (B), and 60 mcg/dose group (C). Dalazatide clinical activity shown as improvement in target lesion for a patient after 4 weeks of treatment from the 60 mcg/dose group (D). Individual TLIGA responses by baseline body weight in the 60 mcg/dose group at Day 32 (E). R = improvement in target lesion; NR = no improvement. Bars indicate mean body weight for each subgroup.

This study was not powered to evaluate clinical efficacy endpoints; however, potential drug-related effects were noted. At baseline, disease severity as assessed by TLIGA (erythema, induration, and scaling for one target psoriatic plaque per subject) was moderate (score of 3) for 23 of the 24 patients, and one patient in the 30 mcg/dose group had severe disease (score of 4). A dose-related trend was observed in the proportion of patients with improvement in the TLIGA at the end of treatment (Day 32) where 1 of 10 patients in the 30 mcg/dose group, and 5 of 10 patients in the 60 mcg/dose group, showed improvements in their target-lesion score relative to baseline (Fig 3A, 3B and 3C). Target lesion improvements occurred as early as Day 15 and continued throughout the 4-week follow up period. An example of target-lesion improvement after dalazatide treatment is shown in Fig 3D.

Data for the 60 mcg/dose group were further evaluated to investigate the effect of body weight on improvement in the TLIGA. Baseline body weight for these 10 patients ranged from 49.2 to 93.5 kg, with a median of 88.8 kg (Table 4). The majority of the patients (*n* = 6) clustered between 88 and 94 kg; 3 patients had a body weight between 73 and 79 kg, and 1 patient weighed 49 kg. All of the patients who weighed less than 88 kg had improvement in the TLIGA, whereas only one higher-weight patient (patient 03–005) responded (Fig 3E). The response of patient 03–005 could not be attributed to unusually high drug exposure; the patient’s C_max_ and AUC_last_ were lower than the mean values at both Day 1 and Day 29.

**Table 4:** Body weight and percent change in PASI score of TLIGA responders and non-responders.

Responders^a^				Non-Responders			
Patient	Baseline body weight (kg)	Dalazatide dose (mcg/kg)	Change in PASI	Patient	Baseline body weight (kg)	Dalazatide dose (mcg/kg)	Change in PASI
01–021	78.7	0.8	−17%	01–001	89.5	0.7	3%
01–028	76.9	0.8	−35%	01–015	88.1	0.7	−30%
03–003	72.7	0.8	−19%	01–023	93.4	0.6	−4%
03–005	92.8	0.6	−34%	03–004	89.4	0.7	−9%
03–006	49.2	1.2	−26%	03–008	93.5	0.6	−6%
Mean±SD	74.1 ± 7.1	–	−26 ± 8.2	Mean±SD	90.8 ± 1.1	–	−9 ± 12.4

^a^ Responders are patients who had improvement in the target lesion at Day 32.

**Fig 4:**
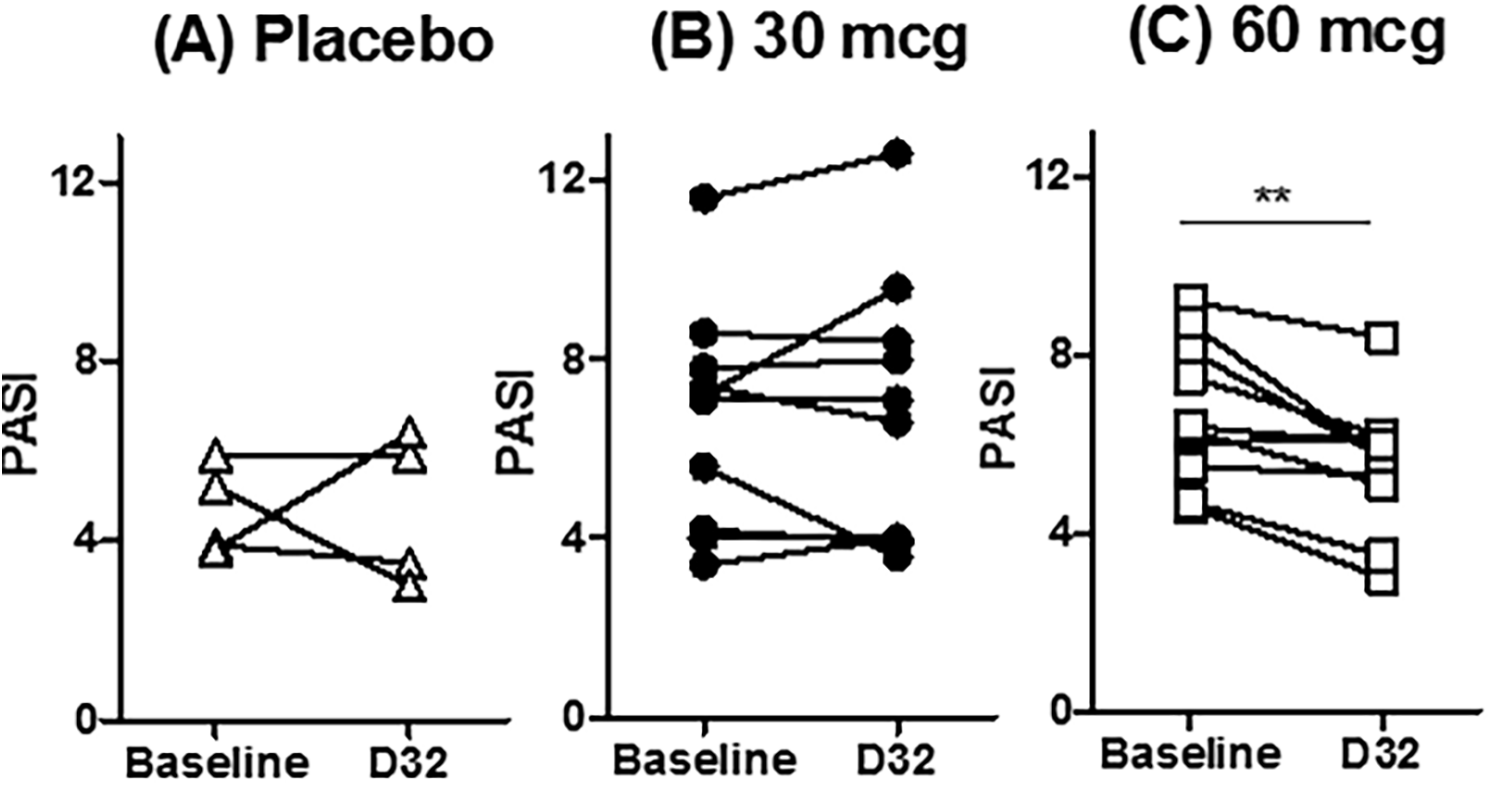
Individual PASI scores at baseline and Day 32. Placebo group (A), 30 mcg/dose group (B), and 60 mcg/dose group (C). *P < 0.01, two-tailed student’s t-test.

**Fig 5:**
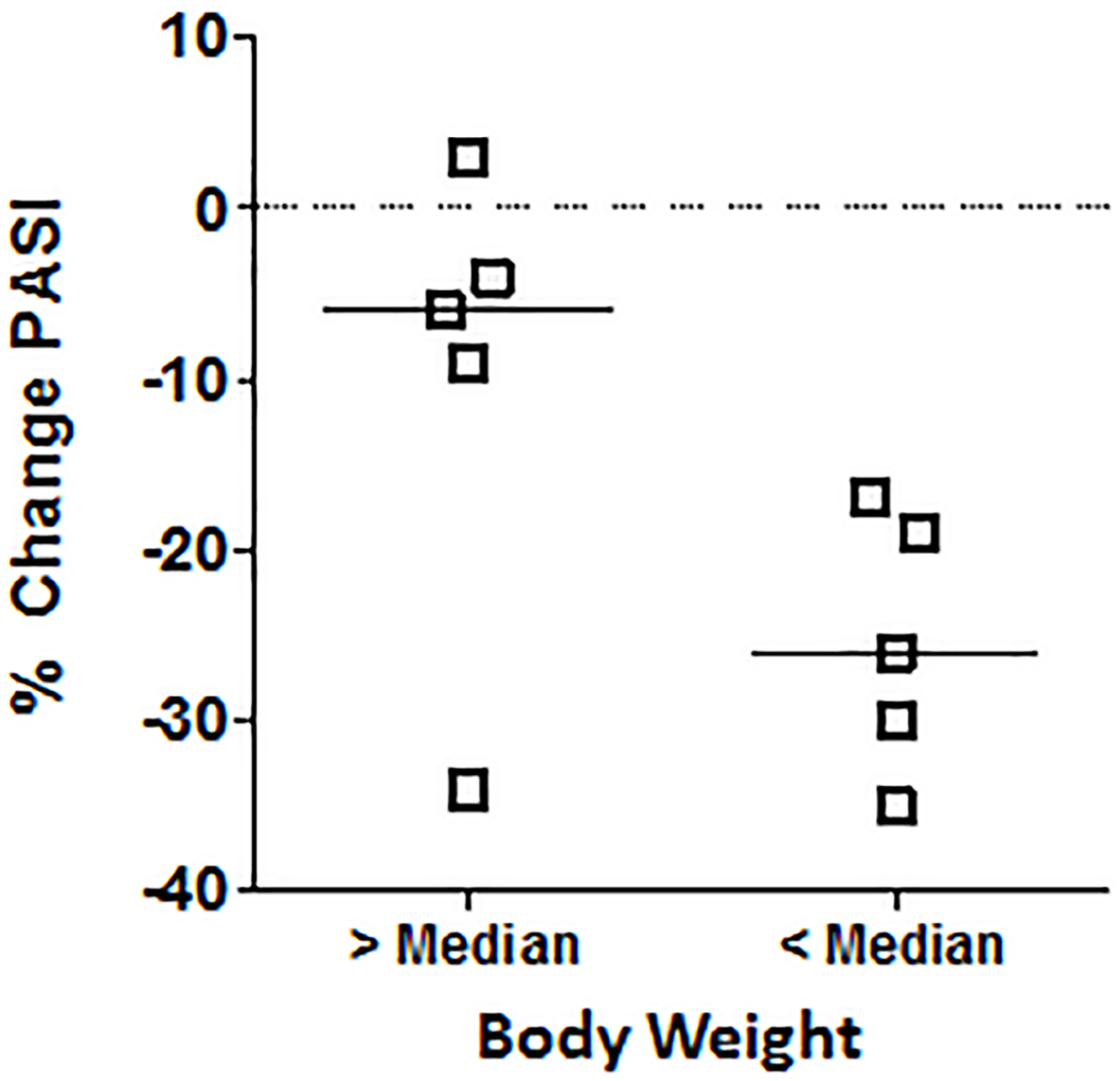
Percent change in PASI score. Change in PASI score for 60 mcg/dose group based on body weight above the median or below the median. Bars indicated mean % change in PASI.

Investigator and patient global assessments of psoriasis also improved, consistent with improvements in the target-lesion score (Table 5). Further, 9 of 10 patients in the 60 mcg/dose group had an absolute reduction in their PASI score between baseline and Day 32, and the mean reduction in PASI score was significant in this group (paired *t*-test, *P* < 0.01) (Fig 4). Data for the 60 mcg/dose group were further evaluated to investigate the effect of body weight on improvement in the PASI. Patients with baseline body weight less than the median for the group had a greater reduction in PASI score between baseline and Day 32 than those with body weight greater than the median (median change of −26% vs −6%, respectively) (Table 4 and Fig 5). Consistent with results observed for the TLIGA, patient 03–005 had a substantial improvement in PASI despite having a relatively heavy body weight.

**Table 5:** Efficacy outcomes.

Assessment	Placebo (*n* = 4)	Dalazatide 30 mcg/dose (*n* = 10)	Dalazatide 60 mcg/dose (*n* = 10)
IGA score improvement, 2-point decrease on Day 32, *n*	0	0	1
IGA score improvement, 1-point decrease on Day 32, *n*	1	2	3
IGA score improvement, 1–2 point decrease on Day 57, *n*	0	0	2
PGA score improvement, unit decrease on Day 32	0	1	1.5
Change in DLQI score from baseline	−2	−5	−3

### Dalazatide treatment reduces the levels of mediators of inflammation associated with psoriasis

**Fig 6:**
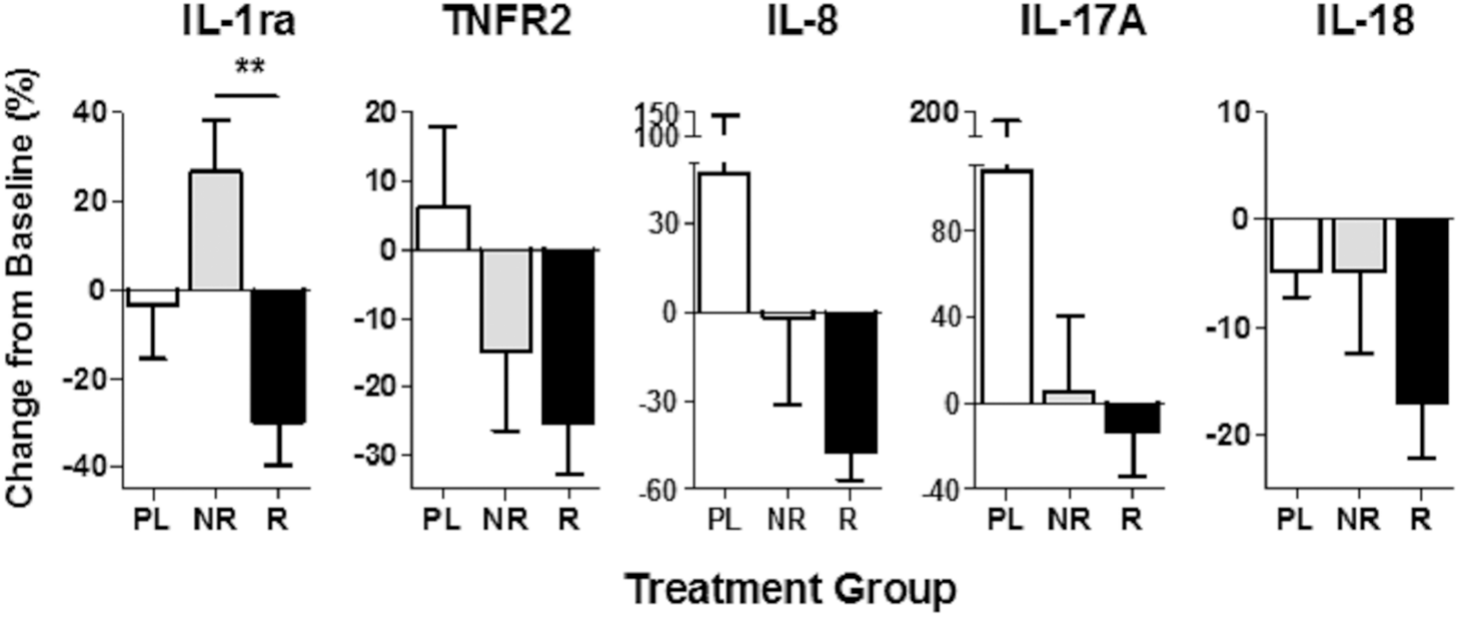
Dalazatide inhibits multiple soluble mediators of inflammation associated with psoriasis. PL = placebo group; R = improvement in target lesion, NR = no improvement in target lesion 60 mcg/dose dalazatide group. Data represent mean ± SEM of the % change from Day 32 relative to baseline (Day 1). **P < 0.01, two-tailed student’s t-test.

The levels of multiple inflammatory cytokines are increased in the blood of patients with psoriasis, and there is a positive correlation between increased blood inflammatory cytokine levels and disease activity [26]. To examine the effects of dalazatide on plasma levels of inflammatory cytokines and other inflammation markers, a combination of multiplex and single-molecule-counting immunoassays were used. Values were analyzed by comparing plasma samples taken at Day 32 to baseline (Day 1 pre first dose) and by grouping samples from dalazatide-treated (60 mcg/dose group) target-lesion responders or non-responders and placebo-treated patients. The plasma levels of the decoy IL-1 receptor antagonist (IL-1ra), soluble TNF-a receptor 2 (TNFR2), IL-8, IL-17A and IL-18 decreased in dalazatide-treated target-lesion responder patients when compared with their plasma baseline on Day 1 (Fig 6). In contrast, the levels of these inflammatory markers were not decreased in the plasma of placebo group patients at the end of treatment relative to baseline levels on Day 1. IL-8 and IL-17A contribute to the pathogenesis of psoriasis by inducing keratinocyte proliferation and are present at increased levels in the skin and circulation of psoriasis patients [27, 28]. Dalazatide treatment reduced IL-8 and IL-17A levels in the plasma of dalazatide-treated target lesion-responders, however, these changes did not reach statistical significance when compared to the treatment effect in the placebo and non-responder groups. TNFR2, IL-1ra, and IL-18 are inflammatory markers that are present at increased levels in a variety of autoimmune diseases, including psoriasis. The reduction of TNFR2 and IL-18 plasma concentrations in dalazatide-treated target-responder patients almost reach statistical significance when compared to the plasma levels patients in the placebo group (TNFR2 *p* = 0.052; IL-18 *p* = 0.087). Notably, the reduction of IL-1ra by dalazatide treatment was significantly higher in target-lesions responders when compared with non-responders. Overall, these data suggest that dalazatide-mediated inhibition of the Kv1.3 channel reduces mediators of inflammation in the blood of psoriasis patients and provide evidence of target engagement.

### Dalazatide treatment reduces the expression of activation markers by memory T cells

**Fig 7:**
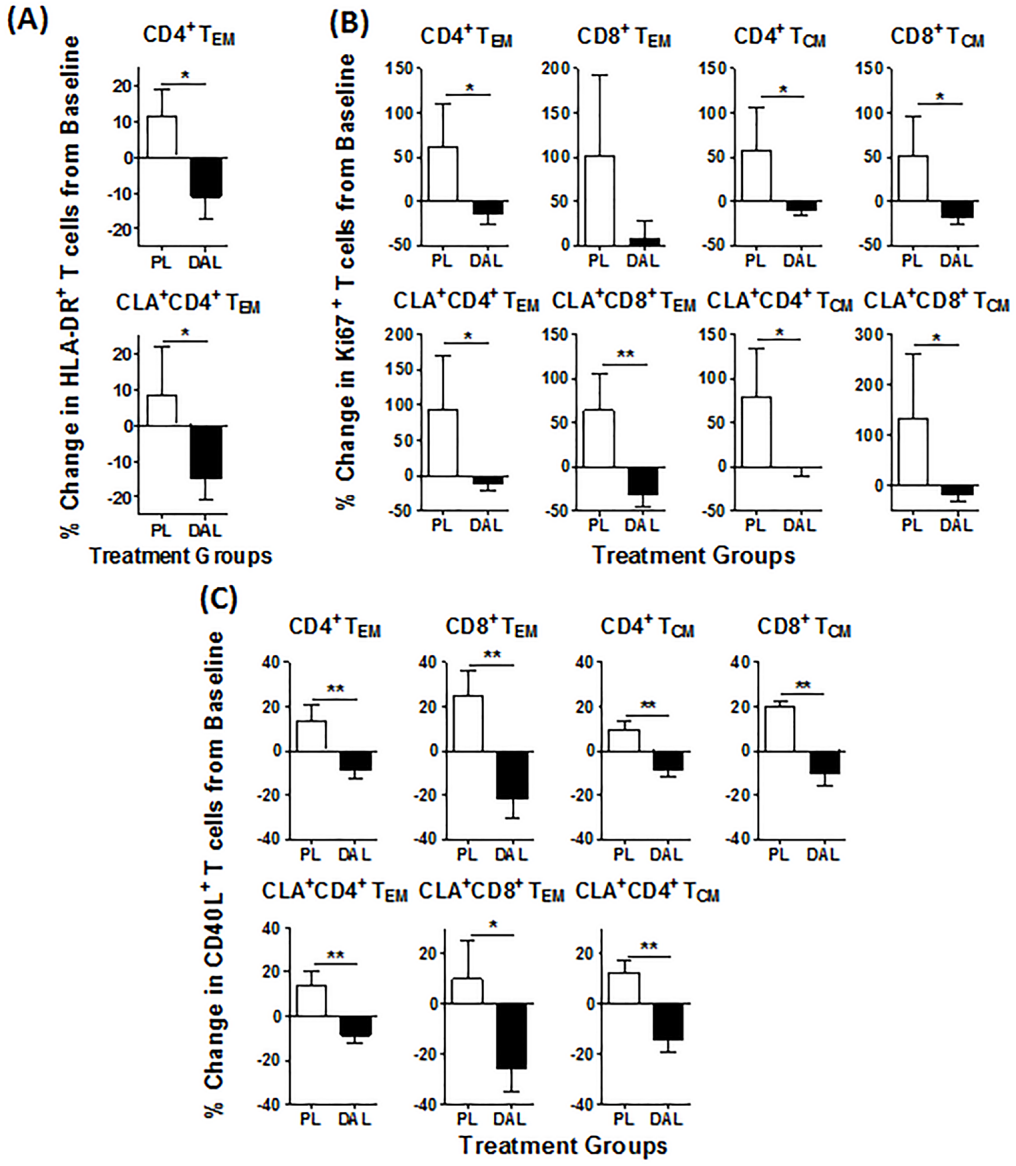
Dalazatide treatment reduces the expression of activation markers HLA-DR, Ki67 and CD40L by memory T cells from psoriasis patients. (A, B) The proportion of CD2+ memory T cells from the peripheral blood from placebo (PAL) and dalazatide (DAL, 60 μχγ/dose) treated patient samples were analyzed for the proportion of the indicated subpopulation and the % change from Day 29 relative to Day 1 calculated. A) HLA-DR expression, B) Ki67 expression, C) CD40L expression after PMA/ionomycin treatment. Samples were collected on Day 1 pre-dose and Day 29 post-dose. The CD40L+ populations as a percentage of CD4+ or CD8+ memory T cells were measured following an *ex vivo* culture of PBMC with 0.2 nM PMA and ionomycin (250 ng/mL) or media alone for 6 hours. The percentage of cells expressing CD40L in response to PMA/ionomycin was calculated as: %CD40L+(stimulated) = %CD40L+(PMA+Ionomycin)-%CD40L+(Media alone). Data represent the mean ± SEM of the % change from Day 29 relative to Day 1. Statistical analysis between placebo and dalazatide groups was performed using one-tailed Student’s t-test. *P < 0.05. **P<0.01

To determine if dalazatide treatment modulates the activation of T cell subsets, PBMC aliquots of patients from the placebo- or 60mcg dalazatide-treated groups were analyzed for the expression of T cell subpopulation specific surface markers such as cutaneous lymphocyte-associated (CLA), CD45RO and CCR7 as well as T cell activation markers. CLA^+^ T cells contribute to the immunepathogenesis of psoriasis and other T cell mediated skin diseases [20]. Marker expression was evaluated on CD2^+^ T cell and CD2^+^ CLA^+^ T cell subpopulations on Day 1 (pre-dose) and Day 29 of the study. CD4^+^ and CD8^+^ effector memory (T_EM_), central memory (T_CM_), and regulatory T cells were analyzed to determine if dalazatide treatment modulated the composition of T cell subsets and reduced the proportion of T cells expressing inflammatory markers. In samples from dalazatide-treated patients (60 mcg/dose group), the percentage of CD4^+^ T_EM_ cells expressing HLA-DR was reduced in Day 29 samples when compared to baseline (Day 1), and this reduction was significantly different from the mean population increases observed in the placebo cohort (Fig 7A). The expression of HLA-DR on activated T cells has been associated with psoriasis and other autoimmune diseases [29]. Similar to the results obtained using global T_EM_ cell populations, an analysis of CLA^+^ T_EM_ cells from patients treated with dalazatide revealed a statistically significant reduction in the percentage of CD4^+^ HLA-DR^+^ cells when compared to placebo group samples. Dalazatide treatment did not affect HLA-DR expression by central memory or regulatory T cell subpopulations when comparing the dalazatide and placebo groups (data not shown).

Ki67 is a nuclear protein that is up-regulated exclusively in proliferating cells. In patients treated with placebo control expression of Ki67 on Day 29 was upregulated from baseline (Day1) by at least 50% on skin homing and general memory T cell subsets (Fig 7B). In contrast, the expression levels of Ki67 on T cells from the 60 mcg dalazatide treatment group on Day 29 were either not affected at all or with the exception of CLA^+^CD8^+^ T_EM_ (30% reduction) slightly reduced (10–18%) when compared to baseline expression. The biggest difference in change of Ki67 expression levels was observed by comparing skin homing CLA^+^CD8^+^ T_EM_ cells from dalazatide and placebo treated patients.

CD40L, a member of the TNF family of molecules, is upregulated in T cells in the circulation of patients with a variety of autoimmune disorders with T cell mediated immunopathologies [30]. Expression of CD40L was evaluated by flow cytometry following *ex vivo* stimulation of the cells with PMA and ionomycin on Day 1 and Day 29. This study was designed to determine the pharmacodynamic effect of dalazatide treatment on *ex vivo* activated T cells from psoriasis patients. PMA/ionomycin treatment increases the expression of Kv1.3 on both effector and central memory T cells. The percentage of CD4^+^ and CD8^+^ T cells expressing CD40L after stimulation was significantly reduced in samples from patients treated with dalazatide, but increased in samples from the placebo group, when comparing the Day 1 pre-dose samples to the Day 29 post-dose samples (Fig 7C). The modulation of cell surface and activation marker expression by dalazatide treatment suggests that the 60 mcg dalazatide treatment dose engaged the Kv1.3 target on CD2+ memory T cells as well as CD2+CLA+ memory T cells homing to the skin of psoriasis patients. The markers identified in this study could potentially serve as pharmacodynamic biomarkers to monitor the biological activity of dalazatide in further clinical studies.

## Discussion

Dalazatide has been evaluated for safety and tolerability in both healthy volunteers as well as patients with plaque psoriasis. No serious adverse events, discontinuations due to adverse events, or deaths occurred during this study and none have occurred during the clinical development program for dalazatide. Likewise, no drug-related trends in serum chemistry, hematology, urinalysis, or electrocardiography were noted in any clinical studies. The most common treatment-related adverse events for dalazatide were instances of paresthesia and hypoesthesia that were somewhat dose dependent. In a previous multiple-ascending-dose study (ClinicalTrials.gov NCT02446340), only a portion of patients in the 5 and 15 mcg/dose cohorts experienced paresthesia or hypoesthesia. Although highly variable within and between individuals, the median duration of paresthesia generally increased with increasing dose up to the 30 mcg/dose group, but not between the 30 and 60 mcg/dose groups. An evaluation of the number of individuals in each cohort that experienced paresthesia each week demonstrated that, in all but the 60 mcg/dose group, the fraction of individuals experiencing paresthesia and the maximum duration of paresthesia decreased over time within a group. Full neurological evaluations from the NCT02446340 trial, including mental status assessment, assessment of cranial nerves II‐XII (field of vision, extraocular movements, pupil function, facial sensation, masseter strength, facial movements, hearing, gag reflex, shoulder shrug, head turn against resistance and tongue movement), sensory examination including distal vibratory sensation, pronator drift, tendon reflexes, gait and coordination, were considered normal both in the presence of ongoing sensory‐related adverse events as well as once these events resolved.

The relationship between potassium channel blockers and paresthesia is well known. Specifically, the transient blockade of potassium channels present in dorsal root ganglia and cutaneous sensory nerve fibers results in functional disturbances that are experienced as paresthesia. Potassium channel blockade is also the mechanism underlying the tingling sensation that results from eating Szechuan peppercorns. The active molecule in the peppercorns, hydroxyl-alpha-sanshool, excites sensory neurons by inhibiting two-pore potassium channels (KCNK3, KCNK9, and KCNK18) [31, 32]. Interestingly, these K2P channels also play a role in autoimmune disease and may represent another potential drug target for disorders involving the central nervous system (reviewed in [33]). Potassium channel blockade can also result in nerve fiber action potential bursting, which is likewise associated with tingling paresthesia [32, 34].

One potassium channel blocking drug that has been tested in humans is the nonselective potassium channel inhibitor, 4-aminopyridine (4-AP, fampridine, dalfampridine). Various formulations of 4-AP have been evaluated for their potential to increase nerve conduction and improve function in spinal cord injury, autoimmune diseases involving the nervous system, and diseases of nerve conduction [35]. The most commonly reported adverse events are paresthesia (perioral and digital), nausea, dizziness, headache, and insomnia. Although infrequent, the most serious toxicity reported in patients treated with 4-AP is seizure [36]. 4-AP and related compounds readily cross the blood-brain barrier [37]. In contrast, dalazatide, like other peptide drugs, is not readily distributed across the intact blood-brain barrier [38, 39]

Several factors in this study impacted the assessment of clinical responsiveness, primarily low baseline BSA and patient bodyweight. Although 9 of 10 patients in the 60 mcg/dose group showed a significant reduction in PASI score, a reduction in PASI is more difficult to achieve in patients with low BSA (< 10%). The PASI is calculated using the BSA and lesion characteristic scores, so when the BSA score is low, changes in the PASI depend almost entirely upon improvement in lesion score. Low baseline BSA therefore results in an underestimation of the degree of improvement [40]. The median baseline BSA for patients in this study was 4 (range: 3 to 7). It was also observed that target-lesion response appeared to be sensitive to body weight; in this study, the body weight of non-responders was significantly higher than that of responders. Improved clinical responsiveness may therefore be achieved with optimized (e.g., weight-based) dosing, a baseline BSA >10%, and a longer duration of treatment.

The mechanism of action of dalazatide is well differentiated from traditional T-cell-directed therapies that block the activation or migration of all T cell populations. Such therapies are broadly immunosuppressive, which can lead to serious adverse effects. Dalazatide is unique in blocking just a subset of the chronically activated T cell population involved in the pathogenesis of autoimmune diseases. Dalazatide treatment may inhibit the production of inflammatory cytokines and therefore may have a broader potential effect than therapies directed at a single cytokine. Furthermore, there is evidence that chronic blockade of the Kv1.3 channel can lead to a dedifferentiation of the T_EM_ cell phenotype into an IL-10 producing immuno-regulatory T cell phenotype [41].

Other psoriasis therapies directed against T_EM_ cells include drugs that prevent co-stimulatory signals needed for T cell activation. These include alefacept, a recombinant human fusion protein consisting of leukocyte-function-associated antigen (LFA) type 3 and IgG1 (approved for the treatment of psoriasis in 2003); and abatacept, a CTLA4-IgG fusion protein approved for the treatment of rheumatoid arthritis. Efalizumab, a monoclonal antibody directed against CD11A that also prevents T cell co-stimulatory signaling, was withdrawn in 2009 due to an increased risk of progressive multifocal leukoencepalopathy. Alefacept is considered one of the safest treatments for psoriasis, but it is effective in only a small proportion of patients [42, 43]. The efficacy of abatacept against psoriasis is still being evaluated in clinical trials [44]. Following subcutaneous administration of 9 doses of 60 mcg dalazatide over 4 weeks to patients with active mild-to-moderate plaque psoriasis, CD4^+^ T_EM_ homing to the skin, were found to express lower levels of the T cell activation marker HLA-DR. In addition, *in vivo* treatment with dalazatide had a pharmacodynamic effect on inhibiting the PMA/ionomycin-induced expression of CD40L by the memory T cell population. The modulation of T cell activation marker expression by dalazatide treatment suggests that dalazatide engaged the Kv1.3 target on memory T cells homing to the skin of psoriasis patients. Despite being a skin disease, systemic inflammation is observed in psoriasis patients and modulation of inflammation markers by dalazatide suggests that dalazatide may have systemic effects. Dalazatide, owing to its specific blockade of the Kv1.3 channel on a subset of T cells, is a first-in-class drug for the treatment of psoriasis. Thus, clinical studies are warranted to further evaluate this therapeutic strategy against psoriasis as well as other autoimmune diseases.

This trial was primarily designed to evaluate the safety, tolerability, and pharmacodynamics of dalazatide. Due to the small patient sample size, short duration of treatment (4 weeks), and mild-to-moderate baseline disease (3% BSA with PASI scores <10), positive clinical responses were not expected. The improvements in target lesion score, PASI, IGA, PGA, and DLQI scores, as well as the decrease in inflammatory cytokines and decrease in T cell activation markers suggest that treatment for longer duration, more frequent dosing, or weight-based dosing may lead to further clinical benefit. This study supports the initiation of mechanism of action studies to evaluate dalazatide in autoimmune disease with T cell immunopathologies mediated by T cells, such as lupus, myositis, multiple sclerosis, vasculitis, and psoriatic arthritis[45].

## Supporting information

S1 CONSORT Checklist CONSORT 2010 checklist. (DOC)

S1 Protocol Redacted study protocol approved by IRB. (PDF)
