# Supplementary material for: Safety and pharmacodynamics of dalazatide, a Kv1.3 channel inhibitor, in the treatment of plaque psoriasis: A randomized phase 1b trial
Source: PLoS One. 2017 Jul 19;12(7):e0180762. doi: 10.1371/journal.pone.0180762 (PMC5516987; doi:10.1371/journal.pone.0180762)
Supplement: S1 Protocol — (PDF) [file pone.0180762.s002.pdf]

SHK-186
PROTOCOL 186-03

A 4 Week Study of the Safety, Tolerability, and Pharmacodynamics of ShK-186 in Active Plaque Psoriasis

OCTOBER 20, 2014

FOR

KINETA ONE, LLC.

[redacted]

INVESTIGATOR/SPONSOR AGREEMENT

I have read the foregoing protocol and agree to conduct the study as described herein.

[redacted]

Date

[redacted]

October 23, 2014
Date

Study Identification

Sponsor	Kineta One, LLC. [redacted]
Sponsor's Study Contact	[redacted]
Medical Monitor	[redacted]

Main Clinical Laboratory [redacted]

Clinical Supply Distributor

Synopsis

Name of Sponsor/Company:

Kineta One, LLC.
219 Terry Ave. N.
Seattle, Washington 98109-5208

Name of Investigational Product:

ShK-186

Name of Active Ingredient:

Kv1.3 channel blocking peptide

Title of Study:

A 4 Week Study of the Safety, Tolerability, and Pharmacodynamics of ShK-186 in Active Plaque Psoriasis

Phase of development:

1b

Study period:

The combined study period will be 8 weeks. Each subject will receive placebo or ShK-186 subcutaneously twice weekly for 4 weeks followed by a 4 week follow-up period. Subjects will be observed in the clinical unit for at least two hours following each dose.

Planned Study Conduct Duration: approximately 5 months total (from enrollment of first subject to completion of follow-up for last subject); 8 weeks for each subject (first dose through End of Study, not including Screening).

Objectives:

Primary:

- assess the safety, tolerability, and immunogenicity of repeated doses of ShK-186 given subcutaneously (SC) in subjects with active plaque psoriasis.

Secondary:

- evaluate ShK-186 drug exposure following repeated doses to subjects;
- evaluate the impact of ShK-186 treatment on psoriasis plaque body surface area involvement (%BSA), Psoriasis Area and Severity Index (PASI) components, and Investigator's global assessment of psoriasis (IGA, 5 point scale) patient global assessment of psoriasis, dermatology life quality index (DLQI) and psoriasis disability index (PDI);
- evaluate the impact of ShK-186 treatment on one unbiopsied target psoriatic plaque;
- evaluate the impact of ShK-186 treatment on psoriatic plaque histopathology (H&E) following biopsy;
- evaluate the impact of ShK-186 treatment on psoriatic plaque inflammatory/proliferative activity by qPCR for gene expression (IFNγ, TNFα, iNOS, IL-4, 8, 10, 17A, 17F, 17A/F, 20, 21,22, 23, CCL20, psoriasin, K16, and other cytokines) and immunohistochemistry for cell activation/populations (KRT16 and Ki67); and mononuclear cell (MNC) infiltrate (CD3, HLA-DR, $CD11c^{+}$, CD68, CD163, Kv1.3) following biopsy;
- evaluate the impact of ShK-186 treatment on plasma/serum biomarkers including IL-17A, IL-17F, IL-17A/F and other cytokines/chemokines (SINGULEX/LUMINEX);

- evaluate impact of ShK-186 on gene expression in whole blood total RNA (Tempus™);
- evaluate the impact of ShK-186 treatment on peripheral blood mononuclear cell populations (CD4/naive TCM, TEM, Treg; CD8/naïve, TCM, TEM) by FACS.

Methodology:

A 4 week, double-blind, placebo controlled study to evaluate two (2) dose levels of ShK-186.

Study design:

This is a double blind, placebo controlled study evaluating two dose levels (30 and 60 µg) of ShK-186. A total of 24 subjects with active plaque psoriasis are to be randomized 5:5:2 to receive either ShK-186 60 µg, ShK-186 30 µg or placebo twice weekly by subcutaneous injection (9 doses total). Potential subjects will be screened to assess their eligibility to enter the study within 30 days prior to the Baseline visit (first dose).

ShK-186 will be prepared for each subject from individual stock vials provided by Kineta. Details of dilution, dose preparation and administration instructions will be provided in a separate study manual.

Number of subjects (planned):

It is estimated that approximately 24 subjects will be enrolled.

Number of Study Centers (planned):

Up to 4 in Canada

Diagnosis and main criteria for inclusion:

Age 18 – 65, inclusive;

Active plaque psoriasis with ≥3% BSA involved;

An adequate number of vulgar psoriatic plaques of at least 2 cm X 2 cm with Target Lesion Investigator Global Assessment scores ≥3, that are not located on the face, scalp, groin, genitals, folds, palms or soles to permit biopsy collection and target lesion assessment.

Inclusion Criteria:

1. Adult male and female subjects, ages 18-65;
2. Active plaque psoriasis with ≥3% BSA involved;
3. An adequate number of vulgar psoriatic plaques of at least 2 cm X 2 cm with Target Lesion Investigator Global Assessment scores ≥3, that are not located on the face, scalp, groin, genitals, folds, palms or soles, to permit:
   a. the longitudinal analysis of a single target lesion throughout the study (no biopsy)
      AND
   b. one of the following:
      i. the collection of two 6 mm biopsies, one pre-dose (Day -7 to -1) and one on Day 32, from the same plaque
      OR
      ii. the collection of four 4 mm biopsies, two pre-dose (Day -7 to -1) from similar plaques (in the same anatomical region) and two on Day 32, from the same two plaques;
4. Weight of 50 – 100 kg;
5. Non-child bearing potential or willingness to use adequate contraception in order to prevent pregnancy from the screening visit until 60 days after the follow-up visit.

Acceptable contraceptive methods for subjects include:

a. barrier methods, such as condom, sponge or diaphragm, combined with spermicide in foam, gel or cream;

b. hormonal contraception (oral, intramuscular implant or transdermal which includes Depo-Provera, Evra and Nuvaring);

c. intrauterine device (IUD).

Non-child bearing potential is defined as:

d. post-menopausal confirmed by FSH test at the screening visit, or;

e. surgically sterile for 60 days prior to the first dose of study drug, during the study and for 60 days after the last dose of study drug by one of the following methods: vasectomy, total hysterectomy, bilateral oophorectomy or bilateral tubal ligation;

6. Subject will be evaluated for latent TB infection with a PPD or a Quantiferon Gold test and Chest X-ray (CXR) within 6 months of the screening visit. Subjects who demonstrates evidence of latent TB infection (either PPD more than or equal to 5 mm of induration or positive Quantiferon Gold, irrespective of Bacillus Calmette-Guerin (BCG) vaccination status and negative CXR findings for active TB, and/or suspicious CXR findings) will not be allowed to participate in the study.
7. Able to communicate and able to provide valid, written informed consent.

Exclusion Criteria:

The following will exclude potential subjects from the study:

1. Erythrodermic, predominantly guttate, exclusively palmar/plantar, or generalized pustular psoriasis;
2. Current drug-induced or aggravated psoriasis (*e.g.*, a new onset of psoriasis or an exacerbation of psoriasis from beta-blockers, calcium-channel blockers, or lithium carbonate);
3. Use of the following concurrent systemic medications: corticosteroids, retinoids, cyclosporine, methotrexate, or biologic agents. The wash out period for these medications is 4 weeks or 5 half-lives prior to baseline, whichever is longer;
4. Use of concurrent topical medications (must be discontinued at least 2 weeks prior to baseline);
5. UVA or UVB therapy within 4 weeks of baseline;
6. The presence of uncontrolled hypertension, uncontrolled diabetes, clinically significant (uncontrolled) cardiovascular disease, uncontrolled asthma or reduced pulmonary capacity, or a history of seizure or other neurologic disorder;
7. Presence or history of pre-existing paresthesia or neuropathy;
8. Abnormalities on neurological exam at screening or baseline;
9. Clinically significant ECG abnormalities, in the opinion of the Investigator;
10. History of any cancer requiring systemic chemotherapy or radiation; individuals with a history of non-melanoma skin cancer, nonrecurring carcinoma in situ treated with laser or cryotherapy, or cervical cancer-in-situ, resected surgically with no evidence of disease, may be accepted on a case by case basis at the discretion of the Investigator;
11. The presence of acute infection or history of acute infection as judged by the Investigator within 7 days of baseline; additionally, oral temperature may not exceed 37.4 °C at baseline;
12. The presence of clinically significant laboratory abnormalities (chemistry panel of 20 analytes [Chem-20; fasted at least 4 hours], complete blood count [CBC], and urinalysis [UA]) as determined by the Investigator;
13. A positive hepatitis screen (Hepatitis BsAg or anti-HCV) or positive Human Immunodeficiency Virus

(HIV) antibody test ;

14. History of treated or untreated TB
15. Any history of anaphylaxis that is important in the view of the Investigator;
16. Participation in another clinical trial with receipt of an investigational product within 90 days of baseline (or 5 half-lives of the previous drug, whichever is longer);
17. History of alcohol abuse that is important in the view of the Investigator;
18. Positive drug screen for amphetamines, barbituates, benzodiazepines, cocaine, cannabis, methamphetamine, methylenedioxymethanphetamine, opiates or phencyclidine
19. Inadequate venous access that would interfere with obtaining blood samples;
20. Positive pregnancy test at screening or at baseline or current lactation (female subjects only);
21. Inability or unwillingness to comply with study restrictions, return for follow up appointments, or other considerations, in the opinion of the Investigator, which would make the candidate unsuitable for study participation.

Investigational product, dosage, and mode of administration:

Following randomization, subjects will receive twice-weekly 0.3 mL SC doses of placebo (normal saline for injection) or ShK-186 as follows (9 total doses):

Group 1: Placebo SC injection twice weekly for 4 weeks (9 doses)

Group 2: 30 μg SC injection of ShK-186 twice-weekly for 4 weeks (9 doses, 270 μg total exposure)

Group 3: 60 μg SC injection of ShK-186 twice-weekly for 4 weeks (9 doses, 540 μg total exposure)

Assessments:

Safety and Immunogenicity:

Vital signs, physical examinations, adverse events (AEs), serum chemistry, hematology, urinalysis, and antibodies to ShK-186**.**

Pharmacokinetics (abbreviated analysis to be performed by a secondary laboratory/Kineta):

- Maximum observed plasma concentration (C_{max})
- Time to maximum plasma concentration (T_{max})
- Trough plasma concentrations pre-dose on Day 15 and Day 57

Disease Activity:

- Change from baseline for Investigator and patient global assessments of psoriasis, DLQI and PDI
- Change from baseline for PASI score components (erythema, induration, and desquamation of skin of head, trunk and extremities)
- Change from baseline for % BSA involved
- Change from baseline in target lesion severity scores for target psoriatic lesion

Biomarker Analysis (to be performed by a secondary laboratory/Kineta):

- Change from baseline for psoriatic plaque histology
- Change from baseline for immune cell populations in psoriatic plaque by immunohistochemistry
- Change from baseline in cytokine expression in psoriatic plaque by qPCR
- Change from baseline for plasma cytokines and chemokines by Multiplex Immuno Assay (Luminex)

- Change from baseline for IL-17A, F and A/F and other cytokines in serum by Single Molecule Counting technology (Singulex)
- Change from baseline in gene expression in whole blood samples (Tempus™)
- Change from baseline in peripheral T cell sub-populations by FACS

Evaluations by Day:

Screening Procedures (Day -30 to -1)

- informed consent;
- medical and medication history including existing paresthesia and neuropathies;
- demographic data;
- weight, height, and BMI;
- routine physical exam (including neurologic exam);
- vital signs (including temperature, respiratory rate, and supine blood pressure and pulse);
- 12-lead ECG;
- assessment of psoriasis enrollment criteria: % BSA involved, adequate number of plaques of appropriate size and severity;
- clinical laboratory evaluations (including Chem-20 [fasted at least 4 hours], CBC, and UA; Appendix H); laboratory evaluations may be repeated if abnormal;
- screens for HIV antibody, screen for hepatitis (HBsAg, anti-HCV), and screen for selected drugs of abuse (Appendix H);
- TB testing if not performed within 6 months of baseline (PPD or QuantiFERON-TB Gold and CXR);
- serum pregnancy test (females only; Appendix H);

Biopsy Procedures (Day -7 to -1)

- Review subject eligibility;
- Photographic documentation of psoriasis-involved body areas (see study manual);
- Identification and photographic documentation of lesional and nonlesional skin to be biopsied (at this visit and on Day 32) (see study manual for selection criteria);
- Identification and photographic documentation of target lesion for on-study assessment (not to be biopsied at any time, see study manual for selection criteria);
- Randomization (must occur before biopsy collection);
- Biopsy of lesional and non-lesional skin (see study manual for procedures and sample handling; subjects will be instructed to abstain from the use of emollients for 24 hours prior to the scheduled biopsy/photography);
- whole blood collection for biomarkers: PBMCs, plasma, serum and whole blood sample for RNA (TEMPUS™).

Baseline (First-Dose) Procedures (Day 1)

Prior to Study Drug Administration

- Review subject eligibility;
- Interim medical and medication history;

- Routine physical exam (including neurologic exam);
- Weight;
- Vital signs (including temperature, respiratory rate, and supine blood pressure and pulse);
- Investigator and patient global assessments of psoriasis[§];
- Patient assessment of quality of life (DLQI)[§];
- Patient assessment of disability (using PDI) [§];
- PASI components[§];
- % BSA involved[§];
- Non-biopsied target lesion assessment[§];
- Whole blood collection for biomarkers: PBMCs, plasma, serum and whole blood sample for RNA (TEMPUS™);
- Clinical laboratory evaluations (including Chem-20 [fasted at least 4 hours], CBC, and UA);
- Baseline plasma sample for study drug concentration;
- Serum sample for assessment of antibodies to ShK-186;
- Urine pregnancy test (for females only, test must be negative to continue participation).

Study Drug Administration

- Study drug will be administered and the start time of study drug administration will be recorded. Postdose study procedures will be based on study drug administration time.

Post-Dose Procedures

At 5 minutes postdose:

- plasma sample for study drug concentration

At 15 minutes postdose:

- plasma sample for study drug concentration.

At 30 minutes postdose:

- vital signs (including temperature, respiratory rate, and supine blood pressure and pulse);
- AE evaluations;
- plasma sample for study drug concentration.

At 1 hour postdose:

- plasma sample for study drug concentration.

At 2 hours postdose:

- vital signs (including temperature, respiratory rate, and supine blood pressure and pulse);
- AE evaluations;
- plasma sample for study drug concentration;

[§] Evaluation can be performed during the biopsy collection visit at the Investigator's discretion.

- whole blood collection for biomarkers: PBMCs, plasma, serum and whole blood sample for RNA (TEMPUS™).

At 4 hours postdose:

- plasma sample for study drug concentration.

Evaluations on Day 2 (Conducted by Telephone):

- medical/medication history;
- AE evaluations.

Follow Up Assessments and Subsequent Doses: (Day 4, 8, 11, 15, 18, 22, 25, 29, 32, 43 and 57)

Some or all of the following procedures will be performed on the various follow-up visits per the study protocol:

- Interim medical and medication history;
- Symptom-directed physical examination;
- AE evaluations;
- Vital signs (including temperature, respiratory rate, and supine blood pressure and pulse);
- Investigator and patient global assessments of psoriasis;
- Patient assessment of quality of life (DLQI);
- Patient assessment of disability (PDI);
- PASI components;
- % BSA involved;
- Non-biopsied target lesion assessment;
- Whole blood collection and processing for plasma, serum and PBMCs for biomarkers;
- Clinical laboratory evaluations (including Chem-20, CBC, and UA);
- Plasma sample for study drug concentration;
- Serum sample for measurement of anti-drug antibodies (ADA);
- Skin biopsy from selected lesion(s) (subjects will be instructed to abstain from the use of emollients for 24 hours prior to the scheduled biopsy/photography);
- Photographic documentation of psoriasis-involved body areas, biopsied lesion(s) and target lesion;
- Serum pregnancy test.

Subject Withdrawal Criteria

The following withdrawal criteria will be utilized to ensure the safety of the subjects participating in the study.

Withdrawal of an individual subject will occur if any one of the following are met:

- Occurrence of an adverse event of Grade 3 or higher (CTCAE v4.0);
    - Grade 3 and greater laboratory abnormalities, that are
        - reproducible and sustainable (repeat laboratory test performed within 24-36 hours), and
        - clinically significant (according to the Investigator's discretion),
    - Grade 3 AEs that are clinically significant but clearly not related to study drug will be

considered individually for withdrawal by the investigator, based on the clinical picture and logistical ability to manage the subject.

- Occurrence of a serious adverse event (SAE);
- Development of paresthesia/peripheral neuropathy symptoms that do not resolve prior to the next dose (at the discretion of the Investigator).

Statistical methods:

Descriptive statistics will be calculated on the safety and efficacy parameters; no formal statistical analyses are planned.

1 TABLE OF CONTENTS, LIST OF TABLES, AND LIST OF FIGURES

1.1 Table of Contents

1 TABLE OF CONTENTS, LIST OF TABLES, AND LIST OF FIGURES 13
1.1 Table of Contents 13
1.2 List of Tables 17
1.3 List of Figures 17
2 LIST OF ABBREVIATIONS AND DEFINITIONS OF TERMS 18
3 INTRODUCTION 20
3.1 ShK-186, Kv1.3 and Autoimmune Disease 20
3.2 ShK-186 Description 20
3.3 Previous Human Experience 21
3.4 Rationale for Dose Selection 21
4 TRIAL OBJECTIVES AND PURPOSE 22
4.1 Primary Objective 22
4.2 Secondary Objective 22
5 INVESTIGATIONAL PLAN 23
5.1 Overall Study Design and Plan: Description 23
5.1.1 Number of Subjects and Centers 23
5.1.2 Estimated Study Duration 23
6 SELECTION AND WITHDRAWAL OF SUBJECTS 24
6.1 Subject Inclusion Criteria 24
6.2 Subject Exclusion Criteria 25
6.3 Subject Withdrawal Criteria 26
6.4 Study Discontinuation 26
7 TREATMENT OF SUBJECTS 28
7.1 Description of Study Drug 28
7.2 Concomitant Medications 28
7.3 Treatment Compliance 28
7.4 Randomization and Blinding 28
8 STUDY DRUG MATERIALS AND MANAGEMENT 30
8.1 Drug Product and Placebo 30
8.2 Study Drug Packaging, Labeling, Storage, Shipping, and Preparation 30

8.3 Study Drug Administration....30
8.4 Study Drug Accountability....31
8.5 Study Drug Handling and Disposal....31
9 STATISTICAL ANALYSES OF SAFETY DATA AND DISEASE ACTIVITY DATA....32
9.1 Safety Data....32
9.2 Pharmacokinetic Data....32
9.3 Disease Activity Data....32
9.4 Sample Size....32
10 DISEASE ACTIVITY ASSESSMENT....33
10.1 Dermatology Quality of Life (DLQI) Questionnaire....33
10.2 Psoriasis Area Severity Index (PASI)....33
10.3 Body Surface Area (BSA)....33
10.4 Psoriasis Disability Index (PDI)....33
10.5 Investigator Global Assessment of Psoriasis....33
10.6 Patient Global Assessment of Psoriasis....33
10.7 Target Lesion Investigator Global Assessment....33
11 ASSESSMENT OF PHARMACOKINETICS....34
11.1 Pharmacokinetic Analysis....34
11.2 Statistical Analysis of Pharmacokinetic Data—Descriptive....34
12 ASSESSMENTS OF IMMUNOGENICITY....35
12.1 Immunoassay – Anti-Drug Antibody....35
12.2 Statistical Analysis of Immunoassay Data—Descriptive....35
13 BIOMARKER ANALYSES....36
13.1 Plasma/PBMC....36
13.2 Skin Biopsies....36
14 STUDY PROCEDURES....37
14.1 Evaluations....37
14.1.1 Screening Procedures (Day -30 to -1)....37
14.1.2 Biopsy Procedures (Pre-dose, Day -7 to -1)....38
14.1.3 Baseline (Day 1, First-Dose) Procedures....38
14.1.4 Evaluations on Day 2....40
14.1.5 Evaluations and procedures on Days 4, 11, 18, and 25....40

14.1.6 Evaluations and procedures on Days 8, 15 and 22 40
14.1.7 Evaluations and procedures on Day 29 41
14.1.8 Evaluations and Procedures on Day 32 and 43 Procedures.......................................42
14.1.9 End of Study Evaluations and Procedures (Day 57) 42
14.2 Safety Evaluations 43
14.2.1 Clinical Laboratory Evaluations..43
14.2.2 Vital Signs 43
14.2.3 AE Evaluations 44
14.2.4 Physical Examinations..44
14.2.5 Neurological Examinations 44
14.3 Sampling for Pharmacokinetic Analysis 44
14.3.1 Pharmacokinetic Sample Collection and Processing...44
14.3.2 Analytical Methodology 44
14.4 Sampling for Biomarker Analyses...44
14.4.1 Blood sample collection for biomarkers...44
14.4.2 Skin biopsies for biomarker evaluation 44
14.4.3 Photographic documentation 44
14.5 Adverse Events 44
14.5.1 Relationship to Study Drug 44
14.5.2 Recording Adverse Events 45
14.5.3 Reporting Serious Adverse Events (SAE)...46
15 DIRECT ACCESS TO SOURCE DATA/DOCUMENTS..48
15.1 Study Monitoring...48
15.2 Audits and Inspections...48
15.3 Institutional Review Board (IRB)) 48
16 ETHICS 49
16.1 Ethical Conduct of the Study...49
16.2 Written Informed Consent 49
16.3 Disclosure 49
17 DATA HANDLING AND RECORD KEEPING 50
17.1 Inspection of Records 50
17.2 Retention of Records 50
18 REFERENCES 51

19 APPENDICES 53
19.1 APPENDIX A: Study Flow Chart 54
19.2 APPENDIX B: Dermatology Life Quality Index (DLQI) 56
19.3 APPENDIX C: Psoriasis Area Severity Index (PASI) 58
19.4 APPENDIX D: Psoriasis Disability Index (PDI) 59
19.5 APPENDIX E: Investigator Global Assessment of Psoriasis 62
19.6 APPENDIX F: Patient Global Assessment of Psoriasis 63
19.7 APPENDIX G: Target Lesion Investigator Global Assessment 64
19.8 APPENDIX H: Clinical Laboratory Evaluations 65

1.2 List of Tables

Table 1. Abbreviations and specialist terms 18
Table 2. Study drug product and placebo 30

1.3 List of Figures

Figure 1: Study schematic for each cohort 23

2 LIST OF ABBREVIATIONS AND DEFINITIONS OF TERMS

The following abbreviations and specialist terms are used in this study protocol.

Table 1: Abbreviations and specialist terms

Abbreviation or specialist term	Explanation
ADA	anti-drug antibodies
AE	adverse event/experience
ALT	alanine aminotransferase (alanine transaminase)
anti-HBc	hepatitis B core antibody
anti-HBs	hepatitis B surface antibody
anti-HCV	hepatitis C virus antibody
API	active pharmaceutical ingredient
AST	aspartate aminotransferase (aspartate transaminase)
BMI	body mass index
BSA	Body Surface Area
CBC	complete blood count (hematology clinical laboratory evaluations)
CD	cluster of differentiation
CFR	Code of Federal Regulations
Chem-20	chemistry panel of 20 analytes
C_{max}	maximum observed plasma concentration
CRF	case report form
CRU	clinical research unit
CTCAE	Common terminology criteria for adverse events
CXR	Chest X-ray
DLQI	Dermatology life quality index
ECG	electrocardiogram
ELISA	enzyme-linked immuno-sorbant assay
FACS	Fluorescence activated cell sorting
FDA	Food and Drug Administration
GCP	Good Clinical Practice
HBsAg	hepatitis B surface antigen
H&E	Hematoxylin and eosin
HIV	Human Immunodeficiency Virus
HLA	Human leukocyte antigen
IB	Investigator Brochure
ICF	Informed Consent Form
IFN	interferon
IGA	Investigator's global assessment
IL	interleukin
iNOS	Inducible nitric oxide synthetase
IRB	Institutional Review Board
Kg	kilogram
mg	milligram

**Abbreviation or specialist term**	**Explanation**
µg	microgram
mL	milliliter
MNC	Mononuclear cell
MTD	maximum tolerated dose
No.	number
PASI	Psoriasis Area Severity Index
PBMC	Peripheral blood mononuclear cell
PDI	Psoriasis disability index
PK	pharmacokinetic
PPD	Purified protein derivative (in reference to TB testing)
Ps	psoriasis
qPCR	quantitative polymerase chain reaction
RNA	Ribonucleic acid
RT-PCR	Reverse-transcriptase polymerase chain reaction
SAE	serious adverse event/experience
SC	subcutaneous
SOP	standard operating procedure
SUSAR	suspected unexpected serious adverse reaction
TB	tuberculosis
T_{max}	time to maximum plasma concentration
T_{CM}	Central memory T cells
T_{EM}	effector memory T cells
TNF	Tumor necrosis factor
UA	urinalysis

3 INTRODUCTION

3.1 ShK-186, Kv1.3 and Autoimmune Disease

ShK-186 targets the Kv1.3 voltage-gated potassium channel (recently reviewed by Chi *et al.* [1]). Interest in this channel derives from its largely restricted expression to effector-memory T (T_{EM}) cells, which mediate tissue damage in autoimmune disease [2,3]. Kv1.3 channel blockers inhibit the activation of effector memory T cells and are effective in animal models of T-cell-mediated autoimmune disease.

During T cell activation, engagement of the T cell receptor by antigen presenting cells results in an influx of calcium into the cytoplasm, initially from the endoplasmic reticulum but subsequently from the extracellular space via the CRAC channel (reviewed by Cahalan and Chandy [4]). Opening of the voltage-gated Kv1.3 or calcium-activated KCa3.1 potassium channels in the T cell membrane and the resulting efflux of potassium ions promotes calcium entry and sustains intracellular calcium at concentrations necessary for T cell activation. Resting T cells express a mixture of both K^+ channels. However, upon activation, naïve and central-memory T (T_{CM}) cells increase expression of the KCa3.1 channel, while T_{EM} cells upregulate and become dependent on Kv1.3 channel expression [3]. In the T_{EM} population, the degree of Kv1.3 expression is a measure of cell activation, and the channel is required for the maintenance of the effector memory cell-phenotype [5].

It has been previously shown that Kv1.3^{HIGH} CD45RA$^-$CCR7$^-$ activated T_{EM} cells are present at sites of inflammation in autoimmune disease, and auto-reactive T cells from subjects with multiple sclerosis, type 1 diabetes, psoriasis (Ps), psoriatic arthritis and rheumatoid arthritis express the Kv1.3^{HIGH} phenotype of activated T_{EM} cells [2,6,7]. In addition, specific Kv1.3-inhibitors have been demonstrated to be effective in numerous animal models of inflammation including adoptive and chronic relapse-remitting experimental autoimmune encephalomyelitis [2,8,9], pristane-induced arthritis [2], the delayed-type hypersensitivity reaction [8–10], allergic contact dermatitis [11], allogeneic kidney transplant [12], spontaneous autoimmune diabetes [2], vascular neointima hyperplasia [13], anti-glomerular basement membrane glomerulonephritis [14], and psoriasis [15].

Kv1.3 is highly expressed in infiltrating T cells in lesional skin of psoriasis and synovium of psoriatic arthritis patients [7,15]. Therefore by blocking the Kv1.3 channel, ShK-186 may have a disease modifying effect in Ps and PsA.

3.2 ShK-186 Description

ShK-186 is a 37 amino acid synthetic peptide derivative of the Stichodactyla toxin [8]. The drug is identified by CAS Registry Number 1081110-69-1.

ShK-186 is a specific inhibitor of the voltage-gated Kv1.3 channel.

The active pharmaceutical ingredient (API) is the ShK-186 peptide acetate salt. The peptide is manufactured using synthetic chemistry under cGMP conditions. The peptide sequence is: (phospho-Tyr)-AEEA-Arg-Ser-Cys-Ile-Asp-Thr-Ile-Pro-Lys-Ser-Arg-Cys-Thr-Ala-Phe-Gln-Cys-Lys-His-Ser-Met-Lys-Tyr-Arg-Leu-Ser-Phe-Cys-Arg-Lys-Thr-Cys-Gly-Thr-Cys-NH2; where AEEA refers to [2-(2-aminoethoxy)] acetic acid. The peptide is cyclized via three disulfide bonds between residues: Cys 3 – Cys 35, Cys 12 – Cys 28, and Cys 17 – Cys 32. The 4442 Da peptide is positively charged and freely soluble in water.

The drug product is provided as a frozen liquid in a single-use borosilicate glass vial with a rubber stopper enclosure (which does not contain latex). The drug product consists of ShK-186 formulated at 1 mg/mL in an isotonic solution of water for injection and USP grade sodium phosphate (10 mM), sodium chloride (0.8%), and Polysorbate 20 (0.05%). The drug product must be diluted in normal saline for injection prior to dose administration.

3.3 Previous Human Experience

In a first-in-human study (186-01), ShK-186 was generally well tolerated by healthy volunteers. Thirty-two subjects received a single dose of ShK-186 or placebo (6:2) in each of 4 cohorts at dose levels of 5, 15, 30 and 60 µg. No death, SAE, AE ≥ Grade 3, or discontinuation due to AE occurred. The vast majority of AEs in this study were mild (Grade 1) in intensity (88%). Paresthesia (tingling sensation around the mouth, hands and feet) and similar AEs occurred in all 18 subjects who received ≥15 µg ShK-186; all these events were mild and resolved spontaneously, usually within 12 hours. There were no other findings with respect to AEs, laboratory values or ECGs during the study. Measurable concentrations of ShK-186/198 were observed in all subjects exposed to ShK-186. ShK-186 exhibited generally dose-proportional single-dose systemic exposure pharmacokinetics (PK).

A 4-week, multiple-ascending dose study of ShK-186 was conducted in healthy volunteers (ShK 186-02) to establish the safety, tolerability and PK profiles of repeated doses in humans. Thirty-two subjects received ShK-186 or placebo (6:2) twice-weekly for 9 doses in each of 4 cohorts at dose levels of 5, 15, 30 and 60 µg. No death, SAE, or AE ≥ Grade 3 occurred during the study. A single subject was withdrawn from the study prematurely after developing viral gastroenteritis. As with the single dose study, the vast majority of the AEs in this study were mild (Grade 1) in intensity. Paresthesia occurred in most subjects who received ≥15 µg ShK-186; all these events were mild and resolved spontaneously, usually within 12 hours of drug administration. At the 15 and 30 µg dose levels, the number or subjects with post-administration paresthesia decreased over time and with continued dosing such that, at the end of 4 weeks, a majority of the subjects in these cohorts no longer reported paresthesia.

For further information, see the current Investigator Brochure.

3.4 Rationale for Dose Selection

In the previous studies (single dose 186-01 and 4-week multiple dose 186-02), the safety and tolerability of ShK-186 was evaluated at doses of 5, 15, 30 and 60 µg.

In these studies, ShK-186 was generally well tolerated at all dose levels. In the 186-01 study, group-median exposures following administration of the 15, 30 and 60 µg dose were 478, 848 and 1420 pg*h/mL, respectively, encompassing the target therapeutic range based on animal efficacy models.

This study is further exploring the 30 and 60 µg doses in subjects with active plaque psoriasis.

4 TRIAL OBJECTIVES AND PURPOSE

4.1 Primary Objective

The primary study objective is to assess safety, immunogenicity, and tolerability of repeat doses of ShK-186 given subcutaneously (SC) in subjects with active plaque psoriasis.

4.2 Secondary Objective

The secondary study objectives are to:

- evaluate ShK-186 drug exposure following repeated doses to subjects;
- evaluate the impact of ShK-186 treatment on psoriasis plaque body surface area involvement (%BSA), Psoriasis Area and Severity Index (PASI) components, Investigator's global assessment of psoriasis (IGA, 5 point scale), patient global assessment of psoriasis, dermatology life quality index (DLQI) and psoriasis disability index (PDI);
- evaluate the impact of ShK-186 treatment on one unbiopsied target psoriatic plaque;
- evaluate the impact of ShK-186 treatment on psoriatic plaque histopathology (H&E) following biopsy;
- evaluate the impact of ShK-186 treatment on psoriatic plaque inflammatory/proliferative activity by qPCR for gene expression (IFNγ, TNFα, iNOS, IL-4, 8, 10, 17A, 17F, 17A/F, 20, 21,22, 23, CCL20, psoriasin, K16, and other cytokines) and immunohistochemistry for cell activation/populations (KRT16 and Ki67) and MNC infiltrate (CD3, HLA-DR, $CD11c^{+}$, CD68, CD163, Kv1.3) following biopsy;
- evaluate the impact of ShK-186 treatment on serum/plasma biomarkers including IL-17A, IL-17F, IL-17A/F and other cytokines/chemokines (SINGULEX/LUMINEX);
- evaluate impact of ShK-186 on gene expression in whole blood total RNA (TEMPUS™);
- evaluate the impact of ShK-186 treatment on peripheral blood mononuclear cell populations (CD4/naive TCM, TEM, Treg; CD8/naïve, TCM, TEM) by FACS.

5 INVESTIGATIONAL PLAN

5.1 Overall Study Design and Plan: Description

This is a double blind, placebo controlled study evaluating two dose levels (30 and 60 µg) of ShK-186. A total of 24 subjects with active plaque psoriasis are to be randomized 5:5:2 to receive either ShK-186 60 µg, 30 µg or placebo twice weekly by subcutaneous injection (9 doses total).

Potential subjects will be screened to assess their eligibility to enter the study within 30 days prior to the Baseline visit (first dose).

5.1.1 Number of Subjects and Centers

It is estimated that approximately 24 subjects will be enrolled at up to 4 centers in Canada.

5.1.2 Estimated Study Duration

Each subject will be screened within 30 days of the Baseline visit (Day 1, Dose 1). Each subject will receive placebo or ShK-186 twice weekly for 4 weeks followed by a 4 week follow-up period.

A study schematic is presented in Figure 1. A study flow chart is presented in Appendix A.

Figure 1. Study schematic for each cohort.

	**Screening***	**Biopsy Collection**	**Dose Administration**	**Evaluations**	**Evaluation and End of Study Procedures**
**Days**	-30 to -1	-7 to -1	1, 4, 8, 11, 15, 18, 22, 25, 29	1, 2 ,4, 8, 11, 15, 18 22, 25, 29, 32, 43	57

*Screening will occur within 30 days of the first dose (Baseline, Day 1) and prior to biopsy collection and randomization. There is no Day 0 in the schedule.

6 SELECTION AND WITHDRAWAL OF SUBJECTS

6.1 Subject Inclusion Criteria

Subjects who meet the following criteria may be included in the study:

1. adult male and female subjects, ages 18-65 inclusive;
2. active plaque psoriasis with $\geq$3% BSA involved;
3. an adequate number of vulgar psoriatic plaques of at least 2 cm X 2 cm with Target Lesion Investigator Global Assessment scores $\geq$3, that are not located on the face, scalp, groin, genitals, folds, palms or soles, to permit:
    a. the longitudinal analysis of a single target lesion throughout the study (no biopsy)

       AND

    b. one of the following:
        i. the collection of two 6 mm biopsies, one pre-dose (Day -7 to -1) and one on Day 32, from the same plaque

           OR

        ii. the collection of four 4 mm biopsies, two pre-dose (Day -7 to -1) from two similar plaques (in the same anatomical region) and two on Day 32, from the same two plaque(s)
4. weight of 50-100 kg;
5. non-child bearing potential or willingness to use adequate contraception in order to prevent pregnancy from the screening visit until 60 days after the follow-up visit.

   Acceptable contraceptive methods for subjects include:

    a. barrier methods, such as condom, sponge or diaphragm, combined with spermicide in foam, gel or cream;
    b. hormonal contraception (oral, intramuscular implant or transdermal which includes Depo-Provera, Evra and Nuvaring);
    c. intrauterine device (IUD).

   Non-child bearing potential is defined as:

    d. post-menopausal confirmed by FSH test at the screening visit, or;
    e. surgically sterile for 60 days prior to the first dose of study drug, during the study and for 60 days after the last dose of study drug by one of the following methods: vasectomy, total hysterectomy, bilateral oophorectomy or bilateral tubal ligation;
6. subjects will be evaluated for latent TB infection with a PPD or a Quantiferon Gold test and CXR within 6 months of the screening visit. Subject who demonstrates evidence of latent TB infection (either PPD more than or equal to 5 mm of induration or positive Quantiferon Gold, irrespective of Bacillus Calmette-Guerin (BCG) vaccination status and negative CXR findings for active TB, and/or suspicious CXR findings) will not be allowed to participate in the study.

7. able to communicate and able to provide valid, written informed consent.

6.2 Subject Exclusion Criteria

The following will exclude potential subjects from the study:

1. erythrodermic, predominantly guttate, exclusively palmar/plantar or generalized pustular psoriasis;
2. current drug-induced or aggravated psoriasis (*e.g.* a new onset of psoriasis or an exacerbation of psoriasis from beta-blockers, calcium channel blockers, or lithium carbonate);
3. use of the following concurrent systemic medications: corticosteroids, retinoids, cyclosporine, methotrexate or biologic agents. The wash out period for all these medications is 4 weeks or 5 half-lives prior to baseline, whichever is longer.
4. use of concurrent topical medications (must be discontinued at least 2 weeks prior to baseline);
5. UVA or UVB therapy within 4 weeks of baseline;
6. the presence of uncontrolled hypertension, uncontrolled diabetes, clinically significant (uncontrolled) cardiovascular disease, uncontrolled asthma or reduced pulmonary capacity or a history of seizure or other neurologic disorder;
7. presence or history of paresthesia or neuropathy;
8. abnormalities on neurologic exam at screening or baseline;
9. clinically significant ECG abnormalities in the opinion of the Investigator;
10. history of any cancer requiring systemic chemotherapy or radiation; individuals with a history of non-melanoma skin cancer, nonrecurring carcinoma in situ treated with laser or cryotherapy or cervical cancer-in-situ, resected surgically with no evidence of disease, may be accepted on a case by case basis at the discretion of the Investigator;
11. the presence of acute infection or history of acute infection as judged by the Investigator within 7 days of baseline; additionally, oral temperature may not exceed 37.4° C at baseline;
12. the presence of clinically significant laboratory abnormalities (chemistry panel of 20 analytes [Chem-20; fasted at least 4 hours], complete blood count [CBC], and urinalysis [UA]) as determined by the Investigator;
13. a positive hepatitis screen (Hepatitis BsAg or anti-HCV) or positive Human Immunodeficiency Virus (HIV) antibody test;
14. history of treated or untreated TB
15. any history of anaphylaxis that is important in the view of the Investigator;
16. participation in another clinical trial with receipt of an investigational product within 90 days of baseline (or 5 half-lives of the previous drug, whichever is longer);
17. history of alcohol abuse that is important in the view of the Investigator;
18. positive drug screen for amphetamines, barbituates, benzodiazepines, cocaine,

cannabis, methamphetamine, methylenedioxymethanphetamine, opiates or phencyclidine

19. inadequate venous access that would interfere with obtaining blood samples;
20. positive pregnancy test at screening or at baseline or current lactation (female subjects only);
21. inability or unwillingness to comply with study restrictions, return for follow up appointments, or other considerations, in the opinion of the Investigator, which would make the candidate unsuitable for study participation.

6.3 Subject Withdrawal Criteria

Subjects who meet all the inclusion criteria and have none of the exclusion criteria will be eligible to be enrolled into the study.

Subjects will be informed that they are free to withdraw from the study at any time and for any reason. The Investigator may remove a subject from the study if, in the Investigator's opinion, it is not in the best interest of the subject to continue the study. Subjects may be withdrawn if there are applicable safety or tolerability concerns on the part of the Investigator. Additionally, withdrawal of an individual subject will occur if any one of the following are met:

- Occurrence of an adverse event of Grade 3 or higher (CTCAE v4.0)
    - Grade 3 and greater laboratory abnormalities, that are
        - reproducible and sustainable (repeat laboratory test performed within 24-36 hours), and
        - clinically significant (according to the Investigator’s discretion),
    - Grade 3 AEs that are clinically significant but clearly <u>not</u> related to study drug will be considered individually for withdrawal by the investigator, based on the clinical picture and logistical ability to manage the subject.
- Occurrence of a serious adverse event (SAE)
- Development of paresthesia/peripheral neuropathy symptoms that do not resolve prior to the next dose (at the discretion of the Investigator).

Notification of discontinuation will be made immediately to the Sponsor. In case of premature discontinuation of study participation, efforts will be made to perform all final study day assessments. The date the subject is withdrawn from the study and the reason for discontinuation will be recorded on the subject’s Case Report Form (CRF). All dropouts will be followed until the resolution of all their AEs or until the unresolved AEs are judged by the Investigator to have stabilized.

Subjects that withdraw after receiving study drug will not be replaced. Subjects that withdraw prior to receiving study drug will be replaced and will receive a new study number.

6.4 Study Discontinuation

The entire study may be discontinued by the Sponsor at its own discretion and for any reason, such as:

- unacceptable safety or tolerability experience;
- medical or ethical reasons affecting the continued performance of the study;
- difficulties in the recruitment of subjects;
- cancellation of drug development.

7 TREATMENT OF SUBJECTS

7.1 Description of Study Drug

See Section 9.

7.2 Concomitant Medications

Excluded systemic medications should be discontinued at least four weeks prior to baseline or five half-lives, whichever is longer.

Topical medications should be discontinued at least two weeks prior to baseline.

Subjects will refrain from participation in another clinical trial with receipt of an investigational product within 90 days of baseline (or 5 half-lives of the previous drug, whichever is longer).

Other agents for the treatment of psoriasis should not be added without prior approval of the Investigator.

7.3 Treatment Compliance

Study drug will be administered at the clinical site by trained study staff.

7.4 Randomization and Blinding

This will be a double-blind, placebo-controlled study. As such, except for the specifically designated unblinded study site clinical and ancillary staff, the Investigator and remaining study site clinical staff will be blinded as to treatment. One or more members of the study site clinical and ancillary staff will remain unblinded during the entire study. These unblinded study site staff will not be involved in any on-study data collection or subject evaluation (*i.e.*, will be only allowed to perform activities such as test material receipt, storage, and handling; unit dose preparation; and/or shipment of laboratory samples).

The original treatment randomization list will be kept secured from the Investigator or Investigator staff until the database is finalized and hard locked. In case of medical emergency (*i.e.*, an adverse reaction that necessitates identification of the test material for the welfare of that subject), the Investigator should contact the Medical Monitor. The Investigator will also have access on site to the randomization code provided by the Sponsor, if the Investigator cannot contact the Medical Monitor. In a safety related event, the Medical Monitor and Sponsor may be unblinded to a subject's treatment in order to assess the event and reporting of the event. The Sponsor must always be notified prior to breaking the blind, EXCEPT in a life-threatening emergency. In these instances, emergency code breaks must be reported to the Sponsor as soon as possible. The time when the study blind was broken, by whom, and the reason why must be documented in the source document and the electronic Case Report Form (eCRF).

Except in a medical emergency, the Investigator or designee and blinded study site clinical staff will remain blinded during the conduct of the study and until such time as all discrepancies in the clinical database are resolved (*i.e.*, at the time of the database lock).

The Investigator will remain blinded as to treatment, for purposes of safety and tolerability data review.

Additionally, study monitors, data management, statisticians, and sponsor or sponsor-contracted personnel involved in review of subject data will remain blinded. Certain designated staff at

CRO and a separate study monitor will be involved in managing study drug and verifying drug accountability and therefore will not be blinded.

8 STUDY DRUG MATERIALS AND MANAGEMENT

8.1 Drug Product and Placebo

ShK-186 is presented as a single-use, preservative-free 0.65 mL vial at a strength of 1 mg/mL.

The formulation buffer contains water for injection, sodium phosphate (10 mM), sodium chloride (0.8%), and Polysorbate 20 (0.05%) at pH 6.0. All excipients meet USP compendial requirements and are not of animal origin.

Prior to administration, the drug product will be diluted by an unblinded member of the study site staff in normal saline for injection to achieve the appropriate concentration for a final administered volume of 0.3 mL. Normal saline to be used for the dilution is the saline used as placebo. More detailed information on the preparation of dosing solutions is presented in the Study Manual.

The Sponsor, or designee, will provide the Investigator with adequate quantities of the study drugs (see Table 2).

Placebo will be Normal Saline Solution for injection provided by the CRO and prepared by an unblinded member of the study site staff.

Table 2. Study drug product and placebo

**Study Drug**	ShK-186	Placebo
**Form**	Solution for subcutaneous injection following dilution in normal saline; packaged in a single-use glass vial	Preservative-free normal saline for injection
**Strength**	1 mg/mL	0.9%
**Manufacturer of Drug Product**	[redacted]	

The lot numbers for the study drugs will be provided to the clinical site by the supplier/manufacturer/distributer/CRO.

8.2 Study Drug Packaging, Labeling, Storage, Shipping, and Preparation

The study drug will be shipped to the site from Sherpa Clinical Packaging or the CRO in vials containing 0.65 mg of ShK-186 at a concentration of 1 mg/mL. The vials will be stored frozen at ≤-20±5°C under secure conditions and protected from light. Details of study drug/placebo packaging, labeling, storage, shipping, and preparation will be provided in a separate Pharmacy Manual.

8.3 Study Drug Administration

Following randomization, subjects will receive twice-weekly 0.3 mL SC doses of placebo (normal saline for injection) or ShK-186 as follows (9 total doses):

Group 1: Placebo SC injection twice weekly for 4 weeks (9 doses)

Group 2: 30 μg SC injection of ShK-186 twice-weekly for 4 weeks (9 doses, 270 μg total exposure);

Group 3: 60 μg SC injection of ShK-186 twice-weekly for 4 weeks (9 doses, 540 μg total exposure);

Appropriate unit doses, as described above, will be administered to the subcutaneous abdominal fat pad of each subject excluding the areas immediately surrounding the navel or waistline. Successive injections will be administered to different sites by rotating between the left/right and upper/lower quadrants of the abdomen. For each dose, the subject's actual time will be recorded in the source documents and transcribed into the CRFs. Further details are provided in the study manual.

8.4 Study Drug Accountability

An unblinded staff member will maintain an accurate record of the receipt of the test materials as shipped by the Sponsor (or designee), including the date received. One copy of this receipt will be returned to the Sponsor (or designee) when the contents of the test material shipment have been verified. In addition, an accurate drug disposition record will be kept, specifying the amount dispensed to each subject and the date of dispensation.

This drug accountability record will be available for inspection at any time by the unblinded monitor. At the completion of the study, the original drug accountability record will be available for review by the Sponsor upon request. Drug inventory and accountability records will be maintained at each site as per GCP/ICH guidelines.

8.5 Study Drug Handling and Disposal

At the completion of the study, all unused drug supplies will be returned to the Sponsor (or designee) or disposed of by the study site, per the Sponsor's (or designee's) written instructions.

9 STATISTICAL ANALYSES OF SAFETY DATA AND DISEASE ACTIVITY DATA

9.1 Safety Data

Safety measures will include vital signs, physical examinations, adverse events, serum chemistry, hematology, urinalysis and evaluation of antibodies to ShK-186. Descriptive statistics will be used in the evaluation of safety parameters. Data on antibodies to ShK-186 will be analyzed as described in Section 12.2. Otherwise, no formal statistical analyses are planned.

9.2 Pharmacokinetic Data

For each group, summary statistics including n, min, max, arithmetic mean and/or median, and standard deviation will be presented for concentration/time, C_{max} and T_{max}. No inferential statistics will be done in this study.

9.3 Disease Activity Data

Disease activity parameters will be measured at various timepoints. Disease activity variables include Investigator and patient global assessment of psoriasis, patient quality of life assessment (using DLQI), patient assessment of disability (using PDI), measurement of psoriasis using PASI component measurements (erythema, induration and desquamation of psoriatic lesions), percent BSA involved and target lesion assessments. Additionally, serum and PBMC biomarkers and lesional biopsy assessments will be performed serially and compared to pre-treatment and non-lesional samples.

Descriptive statistics will be used in the evaluation of disease activity measures. Improvement from baseline in disease activity measures will be evaluated for each timepoint where disease activity parameters are measured.

9.4 Sample Size

The sample size chosen for this study was based upon precedent set by other studies of a similar nature and was not based on power calculations.

10 DISEASE ACTIVITY ASSESSMENT

10.1 Dermatology Quality of Life (DLQI) Questionnaire

The DLQI is a simple 10-question validated questionnaire which will be completed on Day 1 and during study visits on Day 15, Day 32, and Day 57. The questionnaire is provided in Appendix B.

10.2 Psoriasis Area Severity Index (PASI)

Psoriasis Area Severity Index is a scale from 0 to 72 that is regularly used in plaque psoriasis studies. Refer to Appendix C for a complete description of this scale. PASI will be evaluated on Day 1 and during study visits on Day 15, Day 32, and Day 57

10.3 Body Surface Area (BSA)

The BSA affected with psoriasis will be evaluated by a dermatologist. As a reference, the area of the whole palm is counted as 1% BSA.

10.4 Psoriasis Disability Index (PDI)

The PDI is a simple 15-question validated questionnaire which will be completed on Day 1 and during study visits on Day 15, Day 32, and Day 57. The questionnaire is provided in Appendix D.

10.5 Investigator Global Assessment of Psoriasis

The IGA is a 5-point scale to be used by the Investigator to evaluate psoriasis which will be completed on Day 1 and during study visits on Day 15, Day 32, and Day 57. The scale ranges from 0 (“clear”) to 4 (“severe”). The assessment scale is provided in Appendix E.

10.6 Patient Global Assessment of Psoriasis

The patient global assessment is a 10 point scale to evaluate psoriasis severity from 0 (“none”) to 10 (“severe”) which will be completed on Day 1 and during study visits on Day 15, Day 32, and Day 57. The scale is provided in Appendix F.

10.7 Target Lesion Investigator Global Assessment

The target lesion assessment will evaluate a target lesion for erythema, induration and scaling, on a scale of 0 (best) to 4 (worst), using the Target Lesion Investigator Global Assessment scale presented in Appendix G. Target lesion assessments will be completed on Day 1 and during study visits on Day 15, Day 32 and Day 57.

11 ASSESSMENT OF PHARMACOKINETICS

11.1 Pharmacokinetic Analysis

For each subject, the following PK parameters will be calculated after first and final dose administrations (maximum recorded concentration and the time of the maximum recorded concentration), whenever possible, based on the plasma concentration versus time of ShK-186 and its metabolite ShK-198:

C_{max}	Maximum observed plasma concentration.
T_{max}	Time to maximum plasma concentration.

For samples collected prior to dose administration on Day 15 and 57, the trough plasma drug concentration will be reported.

11.2 Statistical Analysis of Pharmacokinetic Data—Descriptive

For each group, summary statistics including n, min, max, arithmetic mean and/or median, and standard deviation will be presented for concentration/time, C_{max} and T_{max}. No inferential statistics will be done in this study.

12 ASSESSMENTS OF IMMUNOGENICITY

12.1 Immunoassay – Anti-Drug Antibody

Serum samples will be collected from each study subject pre-dose on Day 1, on Day 15, Day 29 and End of Study. Serum will be evaluated for binding ADA using an ELISA-based method. Each positive serum sample will be evaluated for ADA specificity by repeating the ELISA in the presence of an excess of ShK-186. Confirmed positive, specific serum samples will be titered by serial dilution, and a numerical titer will be assigned.

12.2 Statistical Analysis of Immunoassay Data—Descriptive

The relationship between dose and antibody titer will be evaluated using the Mann-Whitney test. The relationship between antibody titer and drug C_{max} will be evaluated using Spearman's Rank Order correlation. Any statistical results resulting from exploratory analysis will be interpreted in view of their exploratory nature.

13 BIOMARKER ANALYSES

13.1 Plasma/PBMC

Plasma and PBMC samples will be collected from the whole blood of each study subject at the time of biopsy collection; pre-dose and post-dose on Day 1, Day 15 and Day 29; and during study visits on Day 32, Day 43 and Day 57 (End of study). Plasma will be evaluated for cytokines and chemokines either by multiplex immunoassay (Luminex) or by single molecule counting (Singulex). PBMCs will be evaluated for the frequency of T cell subsets by flow cytometry. Whole blood gene expression analysis will be performed on samples collected using Tempus™ tubes.

13.2 Skin Biopsies

Skin biopsies will be taken from each study subject at: pre-dose (one each from psoriatic lesions and adjacent healthy skin for baseline) and on Day 32 (psoriasis lesions only). At each time, a biopsy of 6 mm will be taken and divided into two 3 mm pieces. If a single lesion of sufficient size is not available, two 4 mm biopsies may be collected from two similar lesions. Biopsies will be analyzed by histology (H&E) and immunohistochemistry for activation/cell-specific markers and by real time quantitative PCR to measure gene expression. Further details of sample collection and storage will be provided in a separate study manual.

14 STUDY PROCEDURES

14.1 Evaluations

Evaluations are summarized in Appendix A. A central laboratory will perform all laboratory evaluations with the following exceptions:

- PK plasma sample analysis will be performed by Chimera Biotec;
- ADA evaluations will be performed by Kineta, Inc.;
- Plasma sample analysis for biomarkers by Luminex and Singulex will be performed by Myriad Genetics Inc. and the Krueger laboratory (Rockefeller University), respectively;
- Skin biopsy samples will be processed and analyzed for histology, immunohistochemistry and gene expression by the Krueger laboratory (Rockefeller University);
- Immunophenotypic evaluation of T cell subsets in PBMC samples will be performed by Kineta, Inc.;
- TEMPUS tubes with whole blood will be processed for RNA and evaluated by QPCR by Kineta Inc.

Every attempt will be made to perform evaluations on the scheduled day and time, as appropriate. Evaluations scheduled to occur on dosing days should be performed within the following post-dose intervals: 5±1 min, 15±5 min, 30±5 min, 1h±10 min, 2h±15 min and 4h±15 min. All of the post-dose procedures should be timed according to the time of dose administration. Evaluations scheduled to occur on non-dosing days through Day 29 should be performed on the day of the scheduled visit. Visits scheduled for beyond Day 29 should be performed within 1 day of the scheduled visit. Deviations from these times should be recorded in the CRF. Missed doses or significant alterations to the timing of a dose are permitted with the prior approval of the Sponsor.

A PPD or QuantiFERON-TB Gold test and chest X-ray will be performed at screening if it was not done within 6 months of Baseline (Day 1). PPD will be performed using 5 tuberculin units (5 TU). Reading will be performed 48 to 72 hours after PPD injection. An induration of 5 mm or more will be considered as positive. If the QuantiFERON-TB Gold test is chosen, it will be performed according to the manufacturer's instructions. Subjects who demonstrate evidence of latent TB infection (either PPD more than or equal to 5 mm of induration or positive Quantiferon Gold, irrespective of BCG vaccination status and negative CXR findings for active TB, and/or suspicious CXR findings) will not be allowed to participate in the study.

Neurological exams will be performed at screening, baseline and at the end of the study. Additional symptom-directed exams may be performed during the dosing period and at the completion of the follow-up period at the Investigator's discretion.

Additional evaluations, such as nerve conduction studies, may be performed in any subject for whom symptoms of paresthesia/peripheral neuropathy or new abnormalities in neurologic examination do not resolve off therapy.

14.1.1 Screening Procedures (Day -30 to -1)

The following Screening procedures will be performed for all potential subjects at a visit

conducted within 30 days of baseline (Day 1, first dose):

1. informed consent;
2. medical and medication history (including pre-existing paresthesia and neuropathies);
3. demographic data;
4. weight, height, and BMI;
5. routine physical exam (including neurologic exam);
6. vital signs (including temperature, respiratory rate, and supine blood pressure and pulse);
7. 12-lead ECG;
8. assessment of psoriasis enrollment criteria: % BSA involved, adequate number of plaques of appropriate size and severity
9. clinical laboratory evaluations (including Chem-20 [fasted at least 4 hours], CBC, and UA; Appendix H); laboratory evaluations may be repeated if abnormal;
10. screens for HIV antibody, screen for hepatitis (HBsAb, anti-HCV), and screen for selected drugs of abuse (Appendix H);
11. TB testing if not performed within 6 months of baseline (PPD or QuantiFERON-TB Gold and CXR);
12. serum pregnancy test (females only; Appendix H).

14.1.2 Biopsy Procedures (Pre-dose, Day -7 to -1)

Within one week of the first dose of study drug, subjects will present to the clinic to obtain a 6 mm punch biopsy of a psoriatic plaque (not the target lesion for assessment), as well as one 6 mm punch biopsy of healthy adjacent skin (at least 2.5 cm away from the lesion). Alternatively, two 4 mm biopsies may be collected if a single 6 mm biopsy cannot be obtained or if subject tolerability would be improved, as judged by the Investigator. Subjects will be instructed to abstain from the use of emollients for 24 hours prior to the scheduled biopsy/photography. Subject eligibility will be reviewed and randomization will occur prior to collection of biopsy tissue.

A target lesion for continued on-study assessment will be identified. Photographic documentation of the target lesion, biopsied lesion(s), location of healthy adjacent skin biopsy and psoriasis-involved body areas will be collected according to procedures described in the study manual.

Whole blood will be collected for biomarker analysis.

Subjects will be randomized.

14.1.3 Baseline (Day 1, First-Dose) Procedures

On Day 1, subjects will report to the study site and the following procedures will be performed.

<u>Prior to Study Drug Administration (Predose Procedures)</u>:

1. interim medical and medication history;
2. routine physical examination (including neurologic exam);

3. weight;
4. vital signs (including temperature, respiratory rate, and supine blood pressure and pulse);
5. Review of subject eligibility
6. patient and Investigator global assessments of psoriasis§;
7. patient quality of life assessment (DLQI)§;
8. patient assessment of disability (using PDI)§;
9. %BSA involved and PASI§;
10. non-biopsied target lesion assessment§;
11. whole blood collection for biomarkers: PBMCs, plasma, serum and whole blood sample for RNA (TEMPUS ™);
12. clinical laboratory evaluations (including Chem-20 [fasted at least 4 hours], CBC, and UA);
13. baseline plasma sample for study drug concentration;
14. serum sample for assessment of antibodies to ShK-186;
15. urine pregnancy test (for females only); for female subjects to continue their participation in the study, the pregnancy test must be negative.

Study Drug Administration:

16. study drug will be administered and the start time of study drug administration will be recorded. Postdose study procedures will be based on study drug administration time.

Post-dose Procedures:

At 5 minutes post dose (±1 min):

17. plasma sample for study drug concentration.

At 15 minutes postdose (±5 min):

18. plasma sample for study drug concentration.

At 30 minutes postdose (±5min):

19. vital signs (including temperature, respiratory rate, and supine blood pressure and pulse);
20. AE evaluations;
21. plasma sample for study drug concentration.

At 1 hour postdose (±10 min):

22. plasma sample for study drug concentration.

At 2 hours postdose (±15 min):

23. vital signs (including temperature, respiratory rate, and supine blood pressure and pulse);

§ Evaluation can be performed during the biopsy collection visit at Investigator's discretion.

24. AE evaluations;
25. plasma sample for study drug concentration;
26. whole blood collection for biomarkers: PBMCs, plasma, serum and whole blood sample for RNA (TEMPUS™).

At 4 hours postdose (±15 min):

27. plasma sample for study drug concentration.

14.1.4 Evaluations on Day 2

On Day 2, study personnel will contact subject via telephone approximately 24 hours postdose and assess:

1. interim medical/medication history;
2. AE evaluations;

14.1.5 Evaluations and procedures on Days 4, 11, 18, and 25

The following procedures will be performed on Days 4, 11, 18, and 25.

<u>Prior to Study Drug Administration (Predose Procedures):</u>

1. medication/medical history;
2. symptom directed physical examination;
3. vital signs (including temperature, respiratory rate, and supine blood pressure and pulse);
4. AE evaluations.

<u>Study Drug Administration (Time Recorded)</u>

<u>Post-dose Procedures:</u>

At 2 hours postdose (±15 min):

5. vital signs (including temperature, respiratory rate, and supine blood pressure and pulse);
6. AE evaluations.

14.1.6 Evaluations and procedures on Days 8, 15 and 22

The following procedures will be performed on Days 8, 15 and 22.

<u>Prior to Study Drug Administration (Predose Procedures):</u>

1. medication/medical history;
2. symptom directed physical examination;
3. vital signs (including temperature, respiratory rate, and supine blood pressure and pulse);
4. AE evaluations;
5. plasma sample for study drug concentration; [Day 15 only]
6. clinical laboratory evaluations (including Chem-20, CBC, and UA; Appendix H);
7. serum sample for assessment of antibodies to ShK-186; [Day 15 only]

8. patient and Investigator global assessments of psoriasis; [Day 15 only]
9. patient quality of life assessment (DLQI); [Day 15 only]
10. patient assessment of disability (PDI); [Day 15 only]
11. %BSA, Psoriasis Area and Severity Index (PASI), and target lesion assessments; [Day 15 only]
12. whole blood collection for biomarkers: PBMCs, plasma, serum and whole blood sample for RNA (TEMPUS™). [Day 15 only]

Study Drug Administration (Time recorded)

Post-dose Procedures:

At 2 hours postdose (±15 min):

13. vital signs (including temperature, respiratory rate, and supine blood pressure and pulse);
14. AE evaluations;
15. whole blood collection for biomarkers: PBMCs, plasma, serum and whole blood sample for RNA (TEMPUS™). [Day 15 only]

14.1.7 Evaluations and procedures on Day 29

The following procedures will be performed on Day 29.

Prior to Study Drug Administration (Predose Procedures):

1. interim medication/medical history;
2. symptom directed physical examination;
3. vital signs (including temperature, respiratory rate, and supine blood pressure and pulse);
4. AE evaluations;
5. plasma sample for study drug concentration;
6. clinical laboratory evaluations (including Chem-20, CBC, and UA);
7. serum sample for assessment of antibodies to ShK-186;
8. whole blood collection for biomarkers: PBMCs, plasma, serum and whole blood sample for RNA (TEMPUS™).

Study Drug Administration (Time Recorded)

Post-Dose Procedures:

At 5 minutes post dose (±1 min):

9. plasma sample for study drug concentration.

At 15 minutes postdose (±5 min):

10. plasma sample for study drug concentration.

At 30 minutes postdose (±5 min):

11. plasma sample for study drug concentration.

At 1 hour postdose (±10 min):

12. plasma sample for study drug concentration.

At 2 hours postdose (±15 min):

13. vital signs (including temperature, respiratory rate, and supine blood pressure and pulse);
14. AE evaluations;
15. plasma sample for study drug concentration
16. whole blood collection for biomarkers: PBMCs, plasma, serum and whole blood sample for RNA (TEMPUS™).

At 4 hours postdose (±15 min):

17. plasma sample for study drug concentration.

14.1.8 Evaluations and Procedures on Day 32 and 43 Procedures

The following procedures will be performed on Days 32 and 43:

1. interim medication/medical history;
2. symptom directed physical examination;
3. vital signs (including temperature, respiratory rate, and supine blood pressure and pulse);
4. AE evaluations;
5. patient and Investigator global assessments of psoriasis; [Day 32 only]
6. patient quality of life assessment (DLQI); [Day 32 only]
7. patient assessment of disability (PDI); [Day 32 only]
8. %BSA, Psoriasis Area and Severity Index (PASI), and target lesion assessments; [Day 32 only]
9. whole blood collection for biomarkers: PBMCs, plasma, serum and whole blood sample for RNA (TEMPUS ™);
10. skin biopsy (subjects will be instructed to abstain from the use of emollients for 24 hours prior to the scheduled biopsy/photography); [Day 32 only]
11. photographic documentation of target lesion that has been assessed throughout, biopsied lesion(s), as well as psoriasis-involved body areas. [Day 32 only]

14.1.9 End of Study Evaluations and Procedures (Day 57)

The following procedures will be performed at the End of Study visit:

1. interim medication/medical history;
2. routine physical examination (including neurologic exam);
3. vital signs (including temperature, respiratory rate, and supine blood pressure and pulse);
4. AE evaluations;
5. patient and Investigator global assessments of psoriasis;

6. patient quality of life assessment (DLQI);
7. patient assessment of disability (PDI);
8. %BSA, Psoriasis Area and Severity Index (PASI) and target lesion assessments;
9. plasma sample for study drug concentration;
10. clinical laboratory evaluations (including Chem-20, CBC, and UA);
11. whole blood collection for biomarkers: PBMCs, plasma, serum and whole blood sample for RNA (TEMPUS ™);
12. photographic documentation of target lesion that has been assessed throughout, biopsied lesion(s), as well as psoriasis-involved body areas (Subjects will be instructed to abstain from the use of emollients for 24 hours prior to the scheduled photography);
13. serum sample for assessment of antibodies to ShK-186;
14. serum pregnancy test (female subjects only);

Subjects that prematurely discontinue from study should complete all assessments for the scheduled visit and all End of Study assessments.

14.2 Safety Evaluations

All subjects who receive study drug will be assessed for safety. The timing of the variables is presented in Appendix A and the variables will include:

- AE evaluations (all AE evaluations will include inquiry regarding the development of numbness/tingling or other symptoms/signs of neuropathy);
- Serious Adverse Events (SAE) evaluations;
- physical examination (including neurologic exam);
- vital signs (including temperature, respiratory rate, and supine blood pressure and pulse);
- clinical laboratory evaluations (including Chem-20, CBC, and UA; Appendix H);
- antibody formation to ShK-186.

14.2.1 Clinical Laboratory Evaluations

Clinical laboratory evaluations (including Chem-20, CBC, and UA).

Serology for hepatitis (see Appendix H), HIV antibody, and selected drugs of abuse will be performed.

Pregnancy tests (females only) will be performed (serum and urine).

14.2.2 Vital Signs

Vital signs (including temperature, respiratory rate, and supine blood pressure and pulse) will be obtained.

Supine blood pressure and pulse will be measured after the subject has been supine for at least 5 minutes.

When vital signs are scheduled at the same time as blood draws, the blood draws will be

obtained at the scheduled time point, and the vitals will be obtained as close to the scheduled time point as possible.

14.2.3 AE Evaluations

Subjects will be asked a non-leading question such as “Have there been any changes in your health status since Screening/since you were last asked?” Subjects will also be encouraged to voluntarily report AEs occurring at any other time during the study. Additionally, subjects will be specifically asked about the development of numbness/tingling or other symptoms of neuropathy.

14.2.4 Physical Examinations

A routine physical examination and symptom-directed examinations will be performed.

14.2.5 Neurological Examinations

A complete neurological examination will be performed as part of screening, baseline and end of study procedures. Additional symptom-directed exams may be performed periodically throughout the study as well as when indicated by adverse events at the discretion of the Investigator.

14.3 Sampling for Pharmacokinetic Analysis

14.3.1 Pharmacokinetic Sample Collection and Processing

Samples for PK analysis of ShK-186 levels will be collected, processed, stored, and shipped according to instructions provided in a separate study manual.

14.3.2 Analytical Methodology

Plasma concentrations of ShK-186 and ShK-198 (an active metabolite of ShK-186) will be determined by Chimera Biotec using a qualified analytical procedure.

14.4 Sampling for Biomarker Analyses

14.4.1 Blood sample collection for biomarkers

Blood used to generate samples of plasma and PBMCs for biochemical and cellular biomarker analyses will be collected, processed, stored and shipped according to instructions provided in a separate study manual.

14.4.2 Skin biopsies for biomarker evaluation

Skin biopsies will be performed, processed, stored and shipped according to instructions provided in a separate study manual and video.

14.4.3 Photographic documentation

A digital camera will be provided to each study site. Photographs of psoriasis-involved body areas, biopsied lesions (prior to biopsy) and target lesions for assessment will be collected. Instructions for capturing and storing images are provided in the study manual.

14.5 Adverse Events

14.5.1 Relationship to Study Drug

The Investigator will make a determination of the relationship of the AE to the study drug using

a 4-category system (not related, possible, probable, definite) according to the following guidelines below:

- **NOT RELATED** = an AE that does not follow a reasonable temporal sequence from administration of the drug and that can be reasonably explained by other factors, including underlying disease, complications, concomitant drugs, or concurrent treatment;
- **POSSIBLE** = an AE that follows a reasonable temporal sequence from the administration of the drug (including the course after withdrawal of the drug) and that cannot be excluded as being possibly caused by the drug (*e.g.*, existence of similar reports attributed to the suspected drug and/or its analogues; reactions attributable to the pharmacological effect of the drug), although other factors such as underlying disease, complications, concomitant drugs, or concurrent treatment are presumable;
- **PROBABLE** = an AE that follows a reasonable temporal sequence from administration of the drug (including the course after withdrawal of the drug) and that can be excluded as being possibly caused by other factors, such as underlying disease, complications, concomitant drugs, or concurrent treatment;
- **DEFINITE** = an AE that follows a reasonable temporal sequence from administration of the drug (including the course after withdrawal of the drug), follows a known or hypothesized cause-effect relationship, and (if appropriate) satisfies the following:
  - positive results obtained in drug sensitivity tests;
  - toxic level of the drug present in blood or other body fluids.

14.5.2 Recording Adverse Events

An AE is defined as any untoward medical occurrence experienced by a patient or healthy subject, whether or not considered drug related by the Investigator. A treatment emergent AE is an AE that is reported after a dose of study drug.

Adverse events/experiences are all:

- unfavorable changes in general condition;
- subjective or objective signs/symptoms;
- concomitant diseases or accidents;
- clinically relevant adverse changes in laboratory parameters observed in a subject in the course of a clinical study.

Adverse events/experiences comprise all disturbances of general health status, subjective and objective disease symptoms (including laboratory abnormalities), and accidents observed in the context of a clinical trial, irrespective of a possible causal relationship with the administration of the trial substance. Events occurring in the framework of a clinical trial during drug-free, and post-study drug periods, under placebo, or in a reference group receiving drug or non-drug therapy, are also to be designated as AEs.

All AEs, whether volunteered, elicited, or noted on physical examination, will be recorded throughout the study (*i.e.*, from the time of first injection on Day 1 until End of Study).

The severity of AEs that are clinical abnormalities, vital signs, and systemic issues will be

categorized according to the criteria presented in the Common Terminology Criteria for Adverse Events v4.0.

14.5.3 Reporting Serious Adverse Events (SAE)

An SAE is any adverse drug experience occurring at any dose that results in any of the following outcomes:

- death;
- a life-threatening adverse drug experience (*i.e.*, places the subject, in the view of the Investigator, at immediate risk of death);
- inpatient hospitalization or prolongation of existing hospitalization;
- a persistent or significant disability/incapacity;
- a congenital anomaly/birth defect;
- important medical event that may require medical or surgical intervention to prevent one of the above outcomes.

Important medical events that may not result in death, be life-threatening, or require hospitalization may be considered SAEs when, based upon appropriate medical judgment, they may jeopardize the patient or subject and may require medical or surgical intervention to prevent one of the outcomes listed in this definition.

All SAEs, related or not, occurring during the study must be reported on an SAE Form within 24 hours of awareness to:

[redacted]

The CRO will notify the Sponsor within one (1) working day of original notification.

Sponsor will process and evaluate all SAEs as soon as the reports are received and will assess the relatedness of each SAE to the study treatment. For each SAE received, the Sponsor will make a determination as to whether the criteria for expedited reporting to relevant regulatory authorities have been met. Innovaderm will manage the expedited reporting of relevant safety information to Health Canada. The sponsor will manage the expedited reporting of relevant safety information to other concerned regulatory agencies in accordance with local laws and regulations.

IRBs and Investigators will be notified of SAEs in compliance with institutional and local requirements.

If a female subject or if a female partner of a male subject becomes pregnant during the study, the subject should inform the study site as soon as possible. No additional treatments shall be administered until a serum pregnancy test is performed and confirmed. Upon confirmation of the pregnancy, the female subject will be discontinued from the treatment portion of the study. Any confirmed pregnancy will be tracked and followed through outcome. While pregnancy itself is

not considered an AE or an SAE, any pregnancy complications or elective termination of a pregnancy for medical reasons will be recorded as an AE and evaluated as a possible SAE. All pregnancies should be reported to the Sponsor and Ethics Committee.

15 DIRECT ACCESS TO SOURCE DATA/DOCUMENTS

The Investigator(s) will permit trial-related monitoring, audits, IRB review, and regulatory inspection(s) by providing direct access to source data/documents.

15.1 Study Monitoring

The CRO will designate both blinded and unblinded Study Monitors who will be responsible for monitoring this clinical trial. The Study Monitors will monitor the study conduct, proper CRF and source documentation completion and retention, and accurate study drug accountability. To this end, the Study Monitors will visit the study site at suitable intervals and be in frequent contact through verbal and written communication. It is essential that the Study Monitors have access to all documents (related to the study and the individual participants) at any time these are requested, with blinding retained at all times for the blinded monitor. In turn, the Study Monitors will adhere to all requirements for subject confidentiality as outlined in the Informed Consent Form (ICF). The Investigator and Investigator's staff will be expected to cooperate with the Study Monitors, to be available during a portion of the monitoring visit to answer questions, and to provide any missing information.

15.2 Audits and Inspections

Audits and inspections will/may be performed according to the study site SOPs and the Good Clinical Practices.

15.3 Institutional Review Board (IRB))

In accordance with 21 CFR 56, the protocol, advertisement, and ICF will be reviewed and approved by the IRB. The Sponsor will supply relevant material for the Investigator or CRO to submit to the IRB for the protocol's review and approval. Verification of the IRB's unconditional approval of the protocol and the written ICF statement will be transmitted to the Investigator.

The IRB will be informed by the Investigator or CRO of subsequent protocol amendments and of serious and unexpected AEs (SUSARs). Approval for protocol amendments will be transmitted in writing to the Investigator. If requested, the Investigator will permit audits by the IRB and regulatory inspections by providing direct access to source data/documents.

The Investigator will provide the IRB with progress reports at appropriate intervals (not to exceed one year) and a Study Progress Report following the completion, termination, or discontinuation of the Investigator's participation in the study.

16 ETHICS

16.1 Ethical Conduct of the Study

The study procedures outlined in this protocol will be conducted in accordance with the U.S. Code of Federal Regulations governing Protection of Human Subjects (21 CFR 50), Financial Disclosure by Clinical Investigators (21 CFR 54), Institutional Review Boards (21 CFR 56), Investigational New Drug Applications (21 CFR 312), and Applications for FDA Approval to Market a New Drug (21 CFR 314), as appropriate. As such, these sections of U.S. Title 21 CFR, along with the applicable International Conference on Harmonization (ICH) Guidelines, are commonly known as Good Clinical Practices, which are consistent with the Declaration of Helsinki and the Tri-Council Policy Statement: Ethical Conduct for Research Involving Humans – 2nd Edition.

16.2 Written Informed Consent

Written informed consent for the study will comply with Health Canada's Requirements for Informed Consent Documents and be obtained from all subjects before protocol-specific procedures are carried out. The ICF will be generated by the CRO and approved (along with the protocol) by the IRB and will be acceptable to the Sponsor.

The Investigator (or designee) will explain the nature of the study and the action of the test product. The subjects will be informed that participation is voluntary and that they can withdraw from the study at any time. In accordance with 21 CFR 50, the informed consent process shall be documented by the use of a written ICF approved by the IRB and will be signed by the subject prior to protocol-specific procedures being performed.

The subject will be given a copy of the signed consent, and the original will be maintained with the subject's records.

16.3 Disclosure

All information provided regarding the study, as well as all information collected/documented during the course of the study, will be regarded as confidential. The Investigator agrees not to disclose such information in any way without prior written permission from the Sponsor.

Any publication of the results, either in part or in total (articles in journals or newspapers, oral presentations, abstracts, etc.) by the Investigator(s) or their representative(s), shall require prior notification and review, within a reasonable time frame, by the Sponsor, and cannot be made in violation of the Sponsor's confidentiality restrictions or to the detriment of the Sponsor's intellectual property rights.

17 DATA HANDLING AND RECORD KEEPING

There will be no alterations in the protocol without agreement between the Sponsor and the Investigator.

There will be no alterations in the protocol affecting subject safety without the express written approval of the Sponsor, Investigator, and IRB.

The results from Screening and data collected during the study will be recorded in the subject's CRF (either paper or electronic CRF). To maintain confidentiality, the subjects/patients will be identified only by numbers and/or initials.

The completed CRFs will be transferred to the Sponsor or designee. Copies of each CRF will be retained by the Investigator. All source documents, records, and reports will be retained by the study site in accordance with 21 CFR 312.62(c).

Data handling and record keeping are further described in the Data Management Plan.

17.1 Inspection of Records

Study records will be made available to appropriate regulatory and monitoring staff according to the clinical site SOPs and GCPs.

17.2 Retention of Records

All primary data, or copies thereof (*e.g.*, laboratory records, CRFs, data sheets, correspondence, photographs, and computer records), which are a result of the original observations and activities of the study and are necessary for the reconstruction and evaluation of any study report, will be retained in the study site archives.

18 REFERENCES

1. Chi V, Pennington MW, Norton RS, Tarcha EJ, Londono LM, Sims-Fahey B, Upadhyay SK, Lakey JT, Iadonato S, Wulff H, Beeton C, Chandy KG. Development of a sea anemone toxin as an immunomodulator for therapy of autoimmune diseases. Toxicon. 2012;59(4):529–46.
2. Beeton C, Wulff H, Standifer NE, Azam P, Mullen KM, Pennington MW, Kolski-Andreaco A, Wei E, Grino A, Counts DR, others. Kv1. 3 channels are a therapeutic target for T cell-mediated autoimmune diseases. Proceedings of the National Academy of Sciences. 2006;103(46):17414–9.
3. Wulff H. The voltage-gated Kv1.3 K+ channel in effector memory T cells as new target for MS. Journal of Clinical Investigation. 2003 Jun 1;111(11):1703–1713.
4. Cahalan MD, Chandy KG. The functional network of ion channels in T lymphocytes. Immunol Rev. 2009 Sep;231(1):59–87. PMID: 19754890
5. Hu L, Pennington M, Jiang Q, Whartenby KA, Calabresi PA. Characterization of the functional properties of the voltage-gated potassium channel Kv1.3 in human CD4+ T lymphocytes. J Immunol. 2007 Oct 1;179(7):4563–4570. PMID: 17878353
6. Rus H, Pardo CA, Hu L, Darrah E, Cudrici C, Niculescu T, Niculescu F, Mullen KM, Allie R, Guo L, Wulff H, Beeton C, Judge SIV, Kerr DA, Knaus H-G, Chandy KG, Calabresi PA. The voltage-gated potassium channel Kv1.3 is highly expressed on inflammatory infiltrates in multiple sclerosis brain. Proc Natl Acad Sci USA. 2005 Aug 2;102(31):11094–11099. PMID: 16043714
7. Raychaudhuri SP, Raychaudhuri SK, Wulff H. Gated Potassium Channel Kv1.3 in Psoriasis and Psoriatic Arthritis: A Therapeutic Target for T Cell-Mediated Autoimmune Diseases [abstract]. Arthritis & Rheumatism. 2009;60(Suppl 10):1434.
8. Beeton C, Pennington MW, Wulff H, Singh S, Nugent D, Crossley G, Khaytin I, Calabresi PA, Chen CY, Gutman GA, others. Targeting effector memory T cells with a selective peptide inhibitor of Kv1. 3 channels for therapy of autoimmune diseases. Molecular Pharmacology. 2005;67(4):1369–1381.
9. Matheu MP, Beeton C, Garcia A, Chi V, Rangaraju S, Safrina O, Monaghan K, Uemura MI, Li D, Pal S, de la Maza LM, Monuki E, Flügel A, Pennington MW, Parker I, Chandy KG, Cahalan MD. Imaging of Effector Memory T Cells during a Delayed-Type Hypersensitivity Reaction and Suppression by Kv1.3 Channel Block. Immunity. 2008 Oct;29(4):602–614.
10. Koo G, Blake J, Talento A, Nguyen M, Lin S, Sirotina A, Shah K, Mulvany K, Hora D, Cunningham P, Wunderler D, McManus O, Slaughter R, Bugianesi R, Felix J, Garcia M, Williamson J, Kaczorowski G, Sigal N, Springer M, Feeney W. Blockade of the voltage-gated potassium channel Kv1.3 inhibits immune responses in vivo. The Journal of Immunology. 1997 Jun 1;158(11):5120 –5128.
11. Azam P, Sankaranarayanan A, Homerick D, Griffey S, Wulff H. Targeting Effector Memory T Cells with the Small Molecule Kv1.3 Blocker PAP-1 Suppresses Allergic Contact Dermatitis. Journal of Investigative Dermatology. 2007 Feb 1;127(6):1419–1429.

12. Grgic I, Wulff H, Eichler I, Flothmann C, Köhler R, Hoyer J. Blockade of T-lymphocyte KCa3.1 and Kv1.3 channels as novel immunosuppression strategy to prevent kidney allograft rejection. Transplant Proc. 2009 Aug;41(6):2601–2606. PMID: 19715983

13. Cheong A, Li J, Sukumar P, Kumar B, Zeng F, Riches K, Munsch C, Wood IC, Porter KE, Beech DJ. Potent suppression of vascular smooth muscle cell migration and human neointimal hyperplasia by KV1.3 channel blockers. Cardiovascular Research. 2011;89(2):282–9.

14. Hyodo T, Oda T, Kikuchi Y, Higashi K, Kushiyama T, Yamamoto K, Yamada M, Suzuki S, Hokari R, Kinoshita M, Seki S, Fujinaka H, Yamamoto T, Miura S, Kumagai H. Voltage-gated potassium channel Kv1.3 blocker as a potential treatment for rat anti-glomerular basement membrane glomerulonephritis. American Journal of Physiology - Renal Physiology. 2010 Dec 1;299(6):F1258 –F1269.

15. Gilhar A, Bergman R, Assay B, Ullmann Y, Etzioni A. The Beneficial Effect of Blocking Kv1.3 in the Psoriasiform SCID Mouse Model. J Invest Dermatol. 2011 Jan;131(1):118–124.

19 APPENDICES

19.1 APPENDIX A: Study Flow Chart

	Screening[7]	Pre-dose	Baseline	Phone	Dose Administration and Evaluation								Evaluation		End of study
**Day**	**-30 to -1**	**-7 to -1**	**1**	**2**	**4**	**8**	**11**	**15**	**18**	**22**	**25**	**29**	**32** +/- 1 day	**43** +/- 1 day	**57** +/- 1 day
**Drug Administration**															
**Dose**			X		X	X	X	X	X	X	X	X			
**Pre-Dose Procedures**															
**Informed consent**	X														
**Inclusion/Exclusion Criteria**	X	X	X												
**Randomization**		X													
**Medical/ Medication History**	X		X	X	X	X	X	X	X	X	X	X	X	X	X
**Demographic data**	X														
**Height/weight/BMI[1]**	X		W												
**Physical exam[2]**	X		X		S	S	S	S	S	S	S	S	S	S	X
**AEs**				X	X	X	X	X	X	X	X	X	X	X	X
**Vital signs**	X		X		X	X	X	X	X	X	X	X	X	X	X
**Safety labs**	X[3]		X[3]			X		X		X		X			X
**Infectious serology[4]**	X														
**TB testing**	X														
**Drug Screening**	X														
**ECG**	X														
**Pregnancy test[5]**	B		U												B
**DLQI**			X					X					X		X
**PDI**			X					X					X		X
**%BSA**	X		X					X					X		X
**PASI**			X					X					X		X
**Investigator Global Assessment of Psoriasis**			X					X					X		X
**–Patient Global Assessment of Psoriasis**			X					X					X		X
**Target lesion identification /assessment**	X	X	X					X					X		X
**Plasma PK**			X					X				X			X
**Serum ADA**			X					X				X			X
**Whole blood biomarker**		X	X					X				X	X	X	X
**Skin biopsy[6]**		X											X		
**Psoriasis photo-documentation**		X											X		X
**Post-dose Procedures**															
**Plasma PK**			6									6			

	**Screening[7]**	**Pre-dose**	**Baseline**	**Phone**	**Dose Administration and Evaluation**								**Evaluation**		**End of study**
**Day**	**-30 to -1**	**-7 to -1**	**1**	**2**	**4**	**8**	**11**	**15**	**18**	**22**	**25**	**29**	**32** +/- 1 day	**43** +/- 1 day	**57** +/- 1 day
**Vital Signs**			2		1	1	1	1	1	1	1	1			
**AEs**			2		1	1	1	1	1	1	1	1			
**Whole blood biomarker**			1					1				1			

1. W=weight only
2. X=full physical exam with neurological component; S=symptom-directed physical exam at the Investigator's discretion
3. Chemistry, Hematology and Urinalysis fasted 4 hours on these days only
4. HIV & Hepatitis
5. B=blood test; U=urine dipstick
6. Baseline = One each from psoriatic lesion and adjacent healthy skin; Day 32 = Psoriasis lesion only
7. Screening will occur within 30 days of the first dose (Baseline, Day 1) and prior to biopsy collection and randomization. There is no Day 0 in the schedule.

19.2 APPENDIX B: Dermatology Life Quality Index (DLQI)

Patient ID #: ___ ___ - ___ ___ ___ Patient Initials: ___ ___ ___

Visit Day: ___________ Visit Date (dd-mmm-yyyy):______________

The aim of this questionnaire is to measure how much your skin problem has affected your life OVER THE LAST WEEK. Please check one box for each question.

1.	Over the last week, how **itchy**, **sore**, **painful** or **stinging** has your skin been?	Very much<br>A lot<br>A little<br>Not at all	☐<br>☐<br>☐<br>☐	
2.	Over the last week, how **embarrassed** or **self conscious** have you been because of your skin?	Very much<br>A lot<br>A little<br>Not at all	☐<br>☐<br>☐<br>☐	
3.	Over the last week, how much has your skin interfered with you going **shopping** or looking after your **home** or **yard?**	Very much<br>A lot<br>A little<br>Not at all	☐<br>☐<br>☐<br>☐	Not relevant ☐
4.	Over the last week, how much has your skin influenced the **clothes** you wear?	Very much<br>A lot<br>A little<br>Not at all	☐<br>☐<br>☐<br>☐	Not relevant ☐
5.	Over the last week, how much has your skin affected any **social** or **leisure** activities?	Very much<br>A lot<br>A little<br>Not at all	☐<br>☐<br>☐<br>☐	Not relevant ☐
6.	Over the last week, how much has your skin made it difficult for you to do any **sport**?	Very much<br>A lot<br>A little<br>Not at all	☐<br>☐<br>☐<br>☐	Not relevant ☐
7.	Over the last week, has your skin prevented you from **working** or **studying**?	Yes<br>No	☐<br>☐	Not relevant ☐
	If "No", over the last week how much has your skin been a problem at **work** or **studying**?	A lot<br>A little<br>Not at all	☐<br>☐<br>☐	

8.	Over the last week, how much has your skin created problems with your **partner** or any of your **close friends** or **relatives**?	Very much<br>A lot<br>A little<br>Not at all	☐<br>☐<br>☐<br>☐	Not relevant ☐
9.	Over the last week, how much has your skin caused any **sexual difficulties**?	Very much<br>A lot<br>A little<br>Not at all	☐<br>☐<br>☐<br>☐	Not relevant ☐
10	Over the last week, how much of a problem has the **treatment** for your skin been, for example by making your home messy, or by taking up time?	Very much<br>A lot<br>A little<br>Not at all	☐<br>☐<br>☐<br>☐	Not relevant ☐

©AY Finlay, GK Khan, April 1992 www.dermatology.org.uk.

Please check you have answered EVERY question. Thank you.

19.3 APPENDIX C: Psoriasis Area Severity Index (PASI)

PASI Scoring (11.12)

Four anatomic sites – head, upper extremities, trunk and lower extremities – are assessed for erythema, induration (plaque thickness), and desquamation (scaling) as seen on the day of the examination. The severity of each sign is assessed using a 5-point scale:

- 0 = No symptoms
- 1 = Slight
- 2 = Moderate
- 3 = Marked
- 4 = Very marked

The area affected by psoriasis within a given anatomic site is estimated as a percentage of the total area of that anatomic site and assigned a numerical value according to the degree of psoriatic involvement as follows:

- 0 = no involvement
- 1 = < 10 %
- 2 = 10 to < 30%
- 3 = 30 to < 50%
- 4 = 50 to < 70%
- 5 = 70 to < 90%
- 6 = 90 to 100 %

Assignments for the following body regions are as follows:

- Neck: include with the head
- Buttocks: include with the lower extremities
- Axillae: include with the trunk
- Genitals: include with the trunk
- The inguinal canal separates the truck and legs anteriorly

The PASI score for each body region is obtained by using the formula

$$PASI = 0.1\,(E_h + I_h + D_h)\,A_h + 0.2\,(E_u + I_u + D_u)\,A_u + 0.3\,(E_t + I_t + D_t)\,A_t + 0.4\,(E_l + I_l + D_l)\,A_l$$

Where *E*, *I*, *D*, and *A* denote erythema, induration, desquamation, and area, respectively, and *h*, *u*, *t*, and *l* denote head, upper extremities, trunk, and lower extremities, respectively.

19.4 APPENDIX D: Psoriasis Disability Index (PDI)

PSORIASIS DISABILITY INDEX

- **Thank you for your help in completing this questionnaire.**
- Please tick one box for every question.
- Every question relates to the **LAST FOUR WEEKS ONLY**.

All questions relate to the LAST FOUR WEEKS.

DAILY ACTIVITIES:

1. How much has your psoriasis interfered with you carrying out work around the house or garden?

Very much ❑
A lot ❑
A little ❑
Not at all ❑

2. How often have you worn different types or colours of clothes because of your psoriasis?

Very much ❑
A lot ❑
A little ❑
Not at all ❑

3. How much more have you had to change or wash your clothes?

Very much ❑
A lot ❑
A little ❑
Not at all ❑

4. How much of a problem has your psoriasis been at the hairdressers?

Very much ❑
A lot ❑
A little ❑
Not at all ❑

5. How much has your psoriasis resulted in you having to take more baths than usual?

Very much ❑
A lot ❑
A little ❑
Not at all ❑

- There are two different versions of questions 6, 7 and 8.
- If you are **at regular work or at school** please answer the first questions **6 - 8**.
- If you are **not at work or school** please answer the second questions **6 - 8**.

All questions relate to the LAST FOUR WEEKS.

WORK OR SCHOOL (if appropriate)

6. How much has your psoriasis made you lose time off work or school over the last four weeks?

Very much ❑
A lot ❑
A little ❑
Not at all ❑

7. How much has your psoriasis prevented you from doing things at work or school over the last four weeks?

Very much ❑
A lot ❑
A little ❑
Not at all ❑

8. Has you career been affected by your psoriasis? e.g. promotion refused, lost a job, asked to change a job.

Very much ❑
A lot ❑
A little ❑
Not at all ❑

IF NOT AT WORK OR SCHOOL: ALTERNATIVE QUESTIONS

6. How much has your psoriasis **stopped you** carrying out your normal daily activities over the last four weeks?

Very much ❑
A lot ❑
A little ❑
Not at all ❑

7. How much has your psoriasis **altered the way** in which you carry out your normal daily activities over the last four weeks?

Very much ❑
A lot ❑
A little ❑
Not at all ❑

8. Has your career been affected by your psoriasis? e.g promotion refused, lost a job, asked to change a job.

Very much ❑
A lot ❑
A little ❑
Not at all ❑

All questions relate to the LAST FOUR WEEKS.

PERSONAL RELATIONSHIPS:

9. Has your psoriasis resulted in sexual difficulties over the last four weeks?
Very much ❑
A lot ❑
A little ❑
Not at all ❑

10. Has your psoriasis created problems with your partner or any of your close friends or relatives?
Very much ❑
A lot ❑
A little ❑
Not at all ❑

LEISURE:

11. How much has your psoriasis stopped you going out socially or to any special functions?
Very much ❑
A lot ❑
A little ❑
Not at all ❑

12. Is your psoriasis making it difficult for you to do any sport?
Very much ❑
A lot ❑
A little ❑
Not at all ❑

13. Have you been unable to use, criticised or stopped from using communal bathing or changing facilities?
Very much ❑
A lot ❑
A little ❑
Not at all ❑

14. Has your psoriasis resulted in you smoking or drinking alcohol more than you would do normally?
Very much ❑
A lot ❑
A little ❑
Not at all ❑

TREATMENT:

15. To what extent has your psoriasis or treatment made your home messy or untidy?
Very much ❑
A lot ❑
A little ❑
Not at all ❑

Please check that you have answered all the questions.

Thank you for your help.

© A Y Finlay 1993. This must not be copied without the permission of the author. PDI Version : Tick-box 1999

19.5 APPENDIX E: Investigator Global Assessment of Psoriasis

**Investigator's Global Assessment**		
**Score**	**Short Descriptor**	**Definition**
0	Clear	No signs of psoriasis; post-inflammatory hyperpigmentation may be present
1	Almost clear	No thickening; normal to pink coloration; no to minimal focal scaling
2	Mild	Just detectable to mild thickening; pink to light red coloration; predominately fine scaling
3	Moderate	Clearly distinguishable to moderate thickening; dull to bright red coloration, moderate scaling
4	Severe	Severe thickening with hard edges; bright to deep dark red coloration; severe/course scaling covering almost all or all lesions

19.6 APPENDIX F: Patient Global Assessment of Psoriasis

PLEASE RATE THE SEVERITY OF YOUR PSORIASIS BY CIRCLING A NUMBER ON THE SCALE BELOW
S'IL VOUS PLAÎT ÉVALUER LA GRAVITÉ DE VOTRE PSORIASIS EN ENCERCLANT UN CHIFFRE SUR L'ÉCHELLE CI-DESSOUS

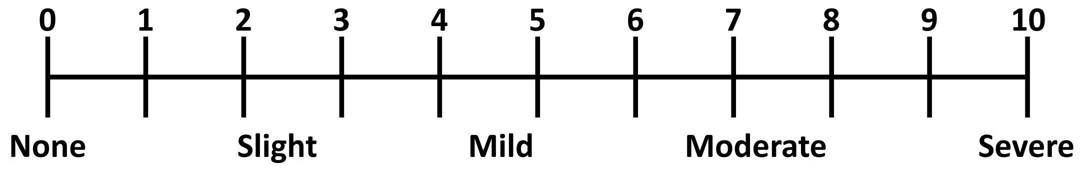

19.7 APPENDIX G: Target Lesion Investigator Global Assessment

**Target Lesion Investigator Global Assessment**		
**Score**	**Short Descriptor**	**Definition**
0	Clear	No signs of psoriasis; post-inflammatory hyperpigmentation may be present
1	Almost clear	No thickening; normal to pink coloration; no to minimal focal scaling
2	Mild	Just detectable to mild thickening; pink to light red coloration; predominately fine scaling
3	Moderate	Clearly distinguishable to moderate thickening; dull to bright red coloration, moderate scaling
4	Severe	Severe thickening with hard edges; bright to deep dark red coloration; severe/course scaling covering almost all or all lesions

19.8 APPENDIX H: Clinical Laboratory Evaluations

Chemistry (Chem-20):
(Fasted 4 hours for screening and baseline visits only)
Albumin
Alkaline Phosphatase
ALT
AST
Blood urea nitrogen
Calcium
Chloride
Cholesterol
Creatinine
Gamma-glutamyl transpeptidase
Glucose
Lactate dehydrogenase
Phosphorus
Potassium
Sodium
Total Bilirubin
Total Protein
Triglycerides
Uric acid

Hematology (CBC):
Hematocrit
Hemoglobin
Mean corpuscular hemoglobin
Mean corpuscular hemoglobin concentration
Mean corpuscular volume
Mean platelet volume
Platelet count
Red blood cell distribution width
Red blood cell count
White blood cell count
White blood cell differential (Percent and Absolute):
- Basophils
- Eosinophils
- Lymphocytes
- Monocytes
- Neutrophils

Other Tests:
HBsAg
Anti-HBc
Anti-HBs
Anti-HCV
HIV Antibody
Pregnancy Test (females only; serum qualitative)
Plasma for study drug concentration
Antibodies to ShK-186

Complete Urinalysis (UA):
Color and appearance
pH and Specific Gravity
Bilirubin
Glucose
Ketones
Leukocytes
Nitrite
Occult blood
Protein
Urobilinogen
Microscopic (including Red Blood Cells and White Blood Cells per High Powered Field)

Drug Screen:
- Alcohol
- Amphetamines
- Barbiturates
- Benzodiazepines
- Cocaine (metabolite)
- Cannibis
- Methamphetamine
- Methylenedioxymethamphetamine
- Opiates
- Phencyclidine
